# Within-patient carbapenem heteroresistance in *klebsiella pneumoniae* associated with a repeat-associated *bla*NDM-1 resistance-module deletion

**DOI:** 10.1016/j.crmicr.2026.100659

**Published:** 2026-08-29

**Authors:** Meiling Jiao, Sibo Wang, Qingyu Meng, Hao Liu, Juntian Jiang, Xuesong Xu, Chao Yan

**Affiliations:** Department of Laboratory Medicine, China-Japan Union Hospital of Jilin University, Changchun, China

**Keywords:** Klebsiella pneumoniae, Carbapenem heteroresistance, *bla*NDM-1, Homologous recombination, Within-host evolution

## Abstract

Carbapenem resistance is often treated as a stable clinical phenotype, although plasmid structural variation can generate closely related isolates with divergent susceptibility profiles. We investigated longitudinal *Klebsiella pneumoniae* isolates recovered from one patient during hospitalization. Resistant KP02 and KP03 and susceptible KP04 belonged to ST37/KL104; KP03 and KP04 were recovered from the same wound specimen and primary culture. Independent sequencing of three colonies from each isolate showed stable within-isolate genomic states and no mutually differing single-nucleotide polymorphisms in the shared callable core. The reassemble data sequences identified a 10,483-bp difference between the IncHI1B/IncFIB(K) multireplicon plasmids Pkp02_1 and Pkp04_1. The interval was delimited by two identical direct repeats containing *sul1* and *qacEΔ1*, and read-level validation, an independent assembly, and structural PCR supported a repeat-associated deletion. An outward-facing PCR product was compatible with a candidate circular intermediate, although its complete circular sequence and abundance remain unresolved. Under antibiotic-free conditions, the module-lacking isolate showed higher growth and final biomass; this association was not assigned uniquely to the deleted module. This single-patient study provides a clinically grounded, hypothesis-generating model for how repeat-associated plasmid rearrangements may contribute to carbapenem phenotypic heterogeneity.

## Highlights

• Longitudinal clinical isolates and independent-colony sequencing supported recent phenotypic diversification within a closely related *Klebsiella pneumoniae* lineage.
• A repeat-associated plasmid deletion removed a *bla*NDM-1-containing resistance module and was supported by reassemble data, read-level validation, and molecular assays.
• The module-lacking isolate showed improved growth under antibiotic-free conditions, whereas the present design does not establish module loss as the sole cause of the fitness difference.
• The repeat architecture was detected in diverse *bla*NDM-1-positive plasmids, supporting its potential relevance to resistance-module instability.

## Graphical abstract

**Figure:**
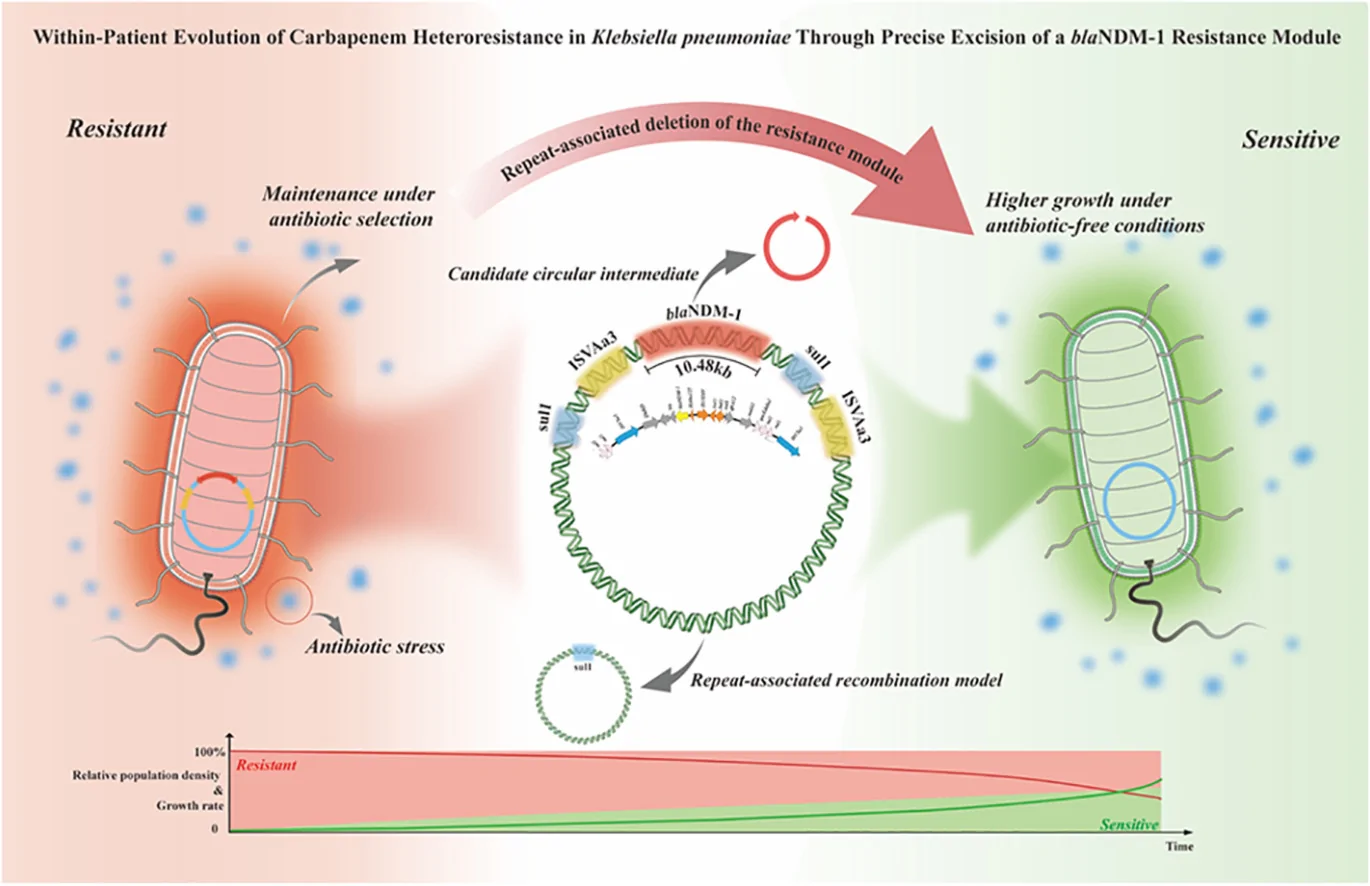

## Introduction

Carbapenem-resistant *Klebsiella pneumoniae* (CRKP) has emerged as one of the most clinically important multidrug-resistant pathogens, driven by the global dissemination of high-risk clonal lineages and mobile resistance determinants (Ernst et al., 2020; Lei et al., 2024). Among these determinants, *bla*NDM genes are of particular concern because they are frequently carried on transferable plasmids and may occur on transposon-associated segments inserted within complex multidrug-resistance regions, thereby limiting therapeutic options and complicating infection control (Wyres et al., 2020; Yang et al., 2021). Despite extensive investigations of CRKP at the population level, the behavior of resistance determinants within individual hosts remains incompletely understood.

Antimicrobial resistance can change under altered selective pressures through plasmid loss, rearrangement, or gene deletion. *bla*NDM genes are frequently carried on transposon-associated mobile segments that can occur near class 1 integron remnants, insertion sequences, and repeated DNA (Zhang et al., 2017; Macesic et al., 2025; Wu et al., 2019). The *qacEΔ1*–*sul1* segment is part of the 3′ conserved region of class 1 integrons; its presence marks a common structural context but does not by itself demonstrate integron activity. Repeated DNA within these regions can provide substrates for rearrangement and may alter resistance phenotypes.

Most evidence for resistance instability comes from in vitro systems or indirect genomic observations (McGann et al., 2012; Tavares et al., 2022; Lopez-Diaz et al., 2022; Liu et al., 2025; Hudson et al., 2014). Clinical studies have focused mainly on resistant subpopulations emerging from susceptible populations, whereas loss of resistance within a closely related lineage is less well characterized (Pereira et al., 2021; Pal and Andersson, 2024; Cho et al., 2025; Nicoloff et al., 2019; Andersson et al., 2019). Direct clinical observations that connect a defined plasmid structural change with coisolated phenotypic variants are therefore uncommon.

We investigated whether a plasmid-borne *bla*NDM-1 region could be lost from a closely related clinical lineage and contribute to coexisting carbapenem-resistant and carbapenem-susceptible isolates. Such heterogeneity may complicate susceptibility assignment when routine testing captures only one colony type from an evolving population.

We analyzed longitudinal *Klebsiella pneumoniae* isolates from one patient to define their clinical provenance, genomic relationship, plasmid structures, and resistance phenotypes. Short- and long-read sequencing, read-level structural validation, molecular assays, and growth experiments were used to evaluate a repeat-associated deletion model. We also assessed the distribution of related repeat architectures among publicly available complete *bla*NDM-1-positive plasmids.

The study provides evidence that a repeat-associated deletion of a *bla*NDM-1*-containing* region coincided with carbapenem phenotypic diversification in one closely related clinical lineage. The findings are hypothesis-generating and require testing in additional clinical collections.

## Methods

### Bacterial isolates

The patient was hospitalized from October 11 through November 19, 2024, for a complicated lower-limb wound infection at the China–Japan Union Hospital of Jilin University, Changchun, China. Wound secretions from the same anatomical site were cultured on three occasions. The October 14 culture yielded *bla*KPC-positive *Klebsiella pneumoniae* KP01 and *Pseudomonas aeruginosa* PA01; the November 1 culture yielded KP02; and the November 5 culture yielded KP03 and KP04. Subsequent mechanistic analyses focused on the clonally related KP02, KP03, and KP04 isolates. Species identification used matrix-assisted laser desorption ionization time-of-flight mass spectrometry (Bruker Daltonics, Germany) according to the manufacturer’s instructions.

### Antimicrobial susceptibility testing

Minimum inhibitory concentrations (MICs) were determined using the broth microdilution method following the Clinical and Laboratory Standards Institute (CLSI) guidelines. The antimicrobial agents tested were imipenem, meropenem, meropenem–vaborbactam, aztreonam–avibactam, ceftazidime–avibactam, piperacillin–tazobactam, ceftazidime, ceftriaxone, cefepime, amikacin, ciprofloxacin, sitafloxacin, colistin, tigecycline, and eravacycline.

Interpretation of the MIC values followed the CLSI 2024 criteria, unless otherwise specified. Breakpoints for colistin and aztreonam–avibactam were interpreted according to the EUCAST 2024 guidelines, whereas tigecycline and eravacycline were interpreted according to the FDA 2023 criteria. *Escherichia coli* ATCC 25,922 and *Pseudomonas aeruginosa* ATCC 27,853 were used as quality control strains.

### Whole-genome sequencing and bioinformatic analysis

Genomic DNA was extracted with the Omega Bio-tek Bacterial DNA Kit (D3350) according to the manufacturer’s instructions. Short-read whole-genome sequencing was performed on the DNBSEQ platform. KP02 and KP04 additionally underwent long-read sequencing, and the corresponding short- and long-read datasets were used for the original hybrid assemblies and for the revised structural analyses. Library preparation and run metadata are reported with the sequencing data submission.

Independent-colony libraries were prepared with the Rapid Plus DNA Lib Prep Kit for Illumina (RK20208) and sequenced in PE150 mode on a NovaSeq X Plus Series platform (Illumina, San Diego, CA, USA). Original short-read data were quality filtered with Sickle, and the original hybrid assemblies were generated with SPAdes 3.12.0. Reassemble data sequences were generated independently by assembling long reads with Flye 2.9.6 and polishing circular contigs twice with the corresponding Illumina reads using Polypolish 0.6.1 after rotation of each replicon between rounds. The KP02 chromosome in the reassemble data assembly was selected after assembly-quality and read-mapping checks as the fixed reference. For the independent-colony datasets, adapters and low-quality bases were removed with Trimmomatic 0.38 using the TruSeq3-PE adapter library, a 4-bp sliding window at mean quality 20, and a minimum read length of 50 bp. Three independently picked colonies from each of KP02, KP03, and KP04 underwent paired-end sequencing. Cleaned reads were analyzed against the fixed reassemble data KP02 chromosome with Snippy 4.6.0; pairwise differences were counted only at positions called as A, C, G, or T in both samples. Within-colony alternative alleles were screened from independently mapped reads using depth, allele-count, strand-support, and quality filters. MLST was assigned with the PubMLST workflow. Plasmid replicons were screened with ABRicate 1.0.1 against the PlasmidFinder database dated 14 January 2025 using minimum identity of 80% and coverage of 60%. Resistance genes and pMLST profiles were determined with ResFinder and the corresponding pMLST workflow, respectively. Detailed parameters and quality-control results are provided in the Supplementary Methods and Tables S3–S6.

### Serial passage

Serial passaging experiments were performed to evaluate the evolutionary dynamics of strain KP02 under reduced antibiotic pressure. KP02 cells were cultured in 5 mL of antibiotic-free LB broth and in LB broth supplemented with sub-inhibitory concentrations of cefoperazone–sulbactam. Cultures were incubated at 37 °C with shaking and passaged every 24 h by transferring 1:1000 into fresh medium. Serial passaging was continued for ten consecutive days. At each passage, bacterial cultures were plated on LB agar plates without antibiotics. Antibiotic susceptibility was monitored by placing imipenem (10 µg), meropenem (10 µg), and cefoperazone–sulbactam (105 µg) disks onto the plates to track phenotypic changes during serial passage.

### PCR validation and quantitative reverse-transcription PCR

Genomic DNA for structural PCR and Sanger validation was extracted with the Omega Bio-tek Bacterial DNA Kit (D3350). Segment PCR used 2 × EasyTaq PCR SuperMix with dye (TransGen Biotech), and outward-facing PCR used 2 × MegaPfu Premix with dye (TOLOBIO, AS231). Amplicons were analyzed by agarose gel electrophoresis, purified with the Omega Bio-tek Gel Extraction Kit (D2500), and subjected to Sanger sequencing. Primer sequences, expected products, cycling conditions, and lane assignments are reported in Supplementary Table S15. Total RNA was isolated with the Omega Bio-tek E.Z.N.A. Bacterial RNA Kit (R6950–01). cDNA was prepared using the FastKing RT Kit with gDNase (Tiangen, KR116), and RT-qPCR used ChamQ Universal SYBR qPCR Master Mix (Vazyme, Q711) with *rpoB* as the reference gene. The original RT-qPCR experiments displayed in Fig. 5E used three independent RNA extractions per isolate, each measured in technical triplicate. A later five-isolate RT-qPCR run was analyzed separately as confirmatory supplementary evidence. Samples without an amplicon in the expected melting-temperature range were classified as target not detected. For graphical display only, target-not-detected wells were represented by a baseline 2^-ΔΔCt value of 1; these display values were not interpreted as relative *bla*NDM-1 expression. Well-level results and assay calls for both datasets are provided in Supplementary Table S16.

### Growth kinetics and in vitro competition assay

Growth kinetics were evaluated by monitoring optical density over time. Overnight cultures were diluted 1:100 in fresh LB broth and incubated at 37 °C with shaking at 180 rpm; OD600 was measured hourly. For the in vitro competition experiment, KP02 and KP04 cultures were adjusted to 0.5 McFarland, mixed 1:1 in LB broth, and sampled every 2 h. Serial dilutions were plated on antibiotic-free LB agar for total counts and on LB agar containing meropenem (4 mg/L) for the carbapenem-resistant fraction. Because differential plating distinguishes resistance phenotype rather than an immutable lineage marker, this assay was interpreted as supportive evidence rather than definitive lineage-specific competition.

### Distribution of direct-repeat architectures among complete *bla*NDM-1-positive plasmids

To assess the distribution of related repeat architectures, a nonredundant collection of complete *bla*NDM-1-positive plasmid sequences was retrieved from the NCBI nucleotide database. Sequences were annotated with Bakta v1.6.1, and related directly repeated *sul1*–*qacEΔ1*–ISVsa3 architectures were visualized. Whole-plasmid relationships were estimated with Mash v2.3 and visualized with iTOL v6; representative structures were compared with Easyfig v2.2.5. This contextual analysis did not estimate the frequency or mechanism of deletion in public plasmids.

### Additional experiments

Conjugation, electroporation, and *Galleria mellonella* infection assays were conducted as described in the Supplementary Methods. Plasmid DNA used for electroporation was purified with the QIAGEN Plasmid Midi Kit. Detailed procedures, controls, and source images are provided in the Supplementary Appendix.

## Results

### Clinical course and bacterial isolation

The period from October 11 through November 19, 2024, represents the complete hospitalization, rather than an inferred duration of infection or an isolate-recovery interval. Wound secretions from the same anatomical site were cultured on October 14, November 1, and November 5. The October 14 culture yielded PA01 and *bla*KPC-positive KP01; the November 1 culture yielded *bla*NDM-1-positive, carbapenem-resistant KP02. On November 5, KP03 and KP04 were recovered as morphologically distinct colonies from the same wound specimen and the same primary culture plate. KP03 was carbapenem resistant and KP04 was carbapenem susceptible. The original wound material and quantitative colony counts are unavailable, so the relative abundance and exact time of emergence of these phenotypes cannot be reconstructed. KP02, KP03, and KP04 belonged to ST37/KL104 and were investigated as a closely related clinical lineage (Fig. 1).

**Fig. 1:**
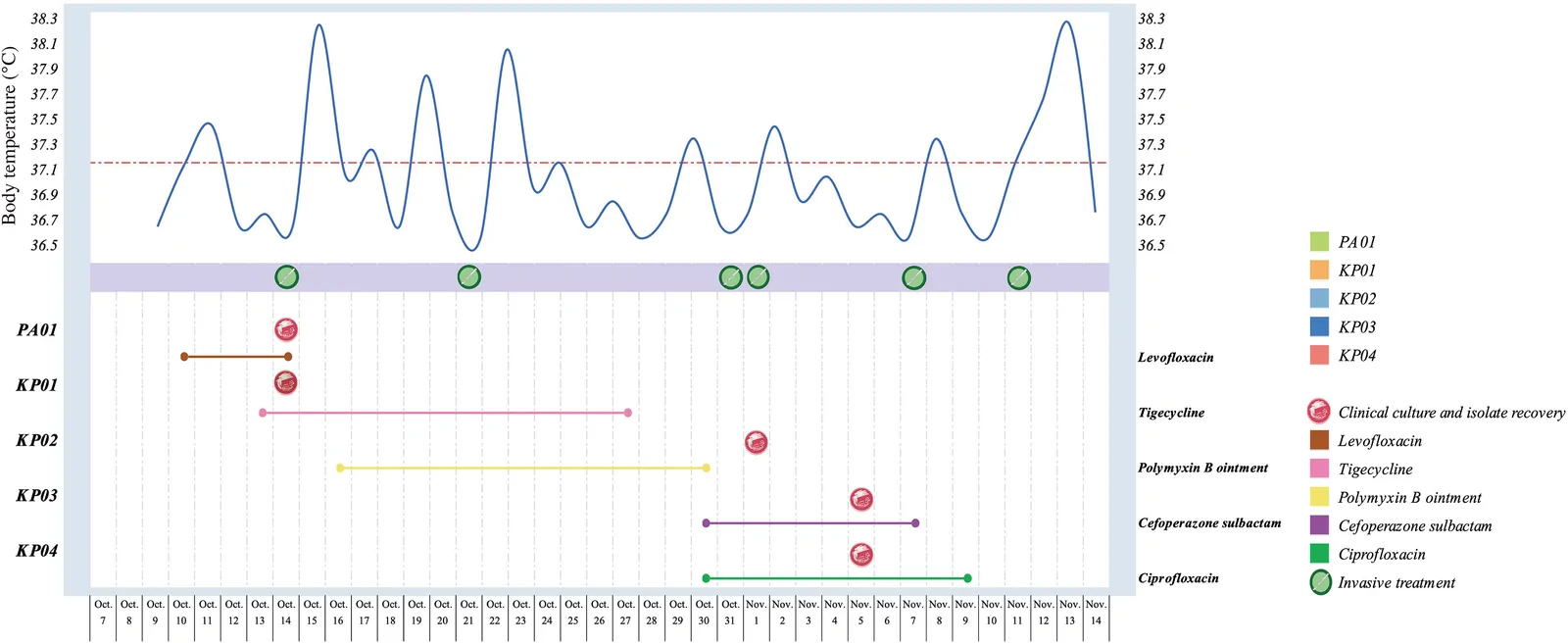
Clinical course and bacterial isolation timeline.

The blue curve shows the recorded body temperature during the period displayed, and the red dashed line indicates 37.2 °C. Colored horizontal lines indicate the antimicrobial treatment periods specified in the key, green symbols indicate invasive procedures, and red culture symbols mark the discrete dates of clinical specimen culture and isolate recovery. Wound secretions from the same right lower-limb site were cultured on October 14, November 1, and November 5, 2024. PA01 and *bla*KPC-positive KP01 were recovered on October 14, and *bla*NDM-1-positive, carbapenem-resistant KP02 was recovered on November 1. On November 5, carbapenem-resistant KP03 and carbapenem-susceptible KP04 were recovered as morphologically distinct colonies from the same wound specimen and the same primary culture plate.

### Antimicrobial susceptibility profiles of KP02 and KP04

KP02 exhibited extensive resistance to multiple antibiotic classes. High-level resistance was observed for several β-lactams (cefepime, ceftazidime, ceftriaxone; MICs >32 μg/mL) and aztreonam (MIC = 64 μg/mL). β-lactam–β-lactamase inhibitor combinations remained ineffective (cefoperazone–sulbactam >128 μg/mL; piperacillin–tazobactam >256 μg/mL). Carbapenem resistance was confirmed (imipenem 8 μg/mL; meropenem 16 μg/mL) and was not reversed by novel inhibitors meropenem–vaborbactam or ceftazidime–avibactam; however, aztreonam-avibactam susceptibility was retained (MIC = 0.125 μg/mL). KP02 also displayed resistance to non-β-lactam agents, including tigecycline, amikacin, fluoroquinolones, colistin, and sulfamethoxazole. In contrast, KP04 exhibited a substantially different profile: restored susceptibility to carbapenems (imipenem <0.06 μg/mL; meropenem <0.03 μg/mL) and to inhibitor combinations (meropenem–vaborbactam, ceftazidime–avibactam, aztreonam–avibactam all <0.03 μg/mL). Non-β-lactam resistance persisted for several agents (Table 1).

**Table 1:** Antimicrobial susceptibility profiles of KP02 and KP04.

Antibiotics	MIC (μg/mL)	
	KP02	KP04
Amikacin	>128	>128
Cefepime	>32	16
Ceftazidime	>32	8
Ceftriaxone	>32	>32
Aztreonam	64	32
Ciprofloxacin	>8	>8
Sitafloxacin	>8	>8
Cefoperazone–Sulbactam	>128	8
Piperacillin–Tazobactam	>256	4
Tigecycline	≥8	≥8
Colistin	>8	>8
Sulfamethoxazole	>16	>16
Imipenem	8	<0.06
Meropenem	16	<0.03
Meropenem–vaborbactam	16	<0.03
Ceftazidime–avibactam	>64	<0.03
Aztreonam–avibactam	0.125	<0.03
Eravacycline	32	0.25

### Clonal relationships revealed by core-genome SNP analysis

KP01 belonged to ST11/KL64, whereas KP02, KP03, and KP04 belonged to ST37/KL104. Three independently selected colonies from each of KP02, KP03, and KP04 were sequenced. All within-parent comparisons contained 0 SNPs, and no mutually differing SNP was detected in the shared callable core across the nine colonies. No intermediate-frequency SNV met the prespecified filtering criteria. *bla*NDM-1 was retained in all KP02 and KP03 colonies and absent from all KP04 colonies. KK167, an additional assembly from the broader 2024 institutional material, differed from each index isolate at approximately 3018 pairwise callable positions across approximately 5.054 Mb. The other available comparison assemblies were ST11 or ST15. This broader comparison reduced the likelihood that the close KP02–KP04 relationship reflected local co-infection with a closely matching background (Fig. 2; Supplementary Tables S7 and S8).

**Fig. 2:**
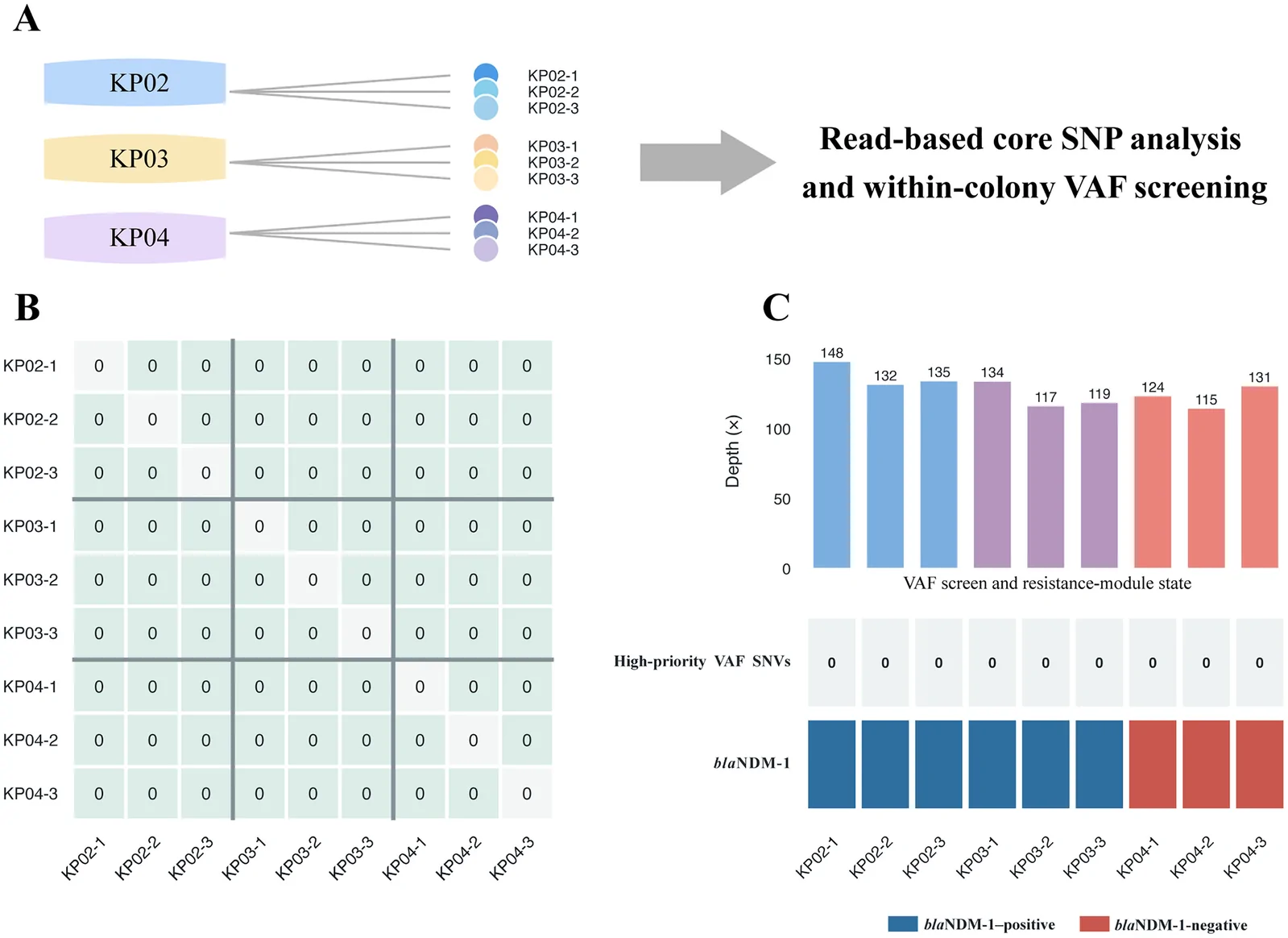
Independent-colony sequencing and within-colony variant screening. (A) Three independently picked colonies from each primary isolate, KP02, KP03, and KP04, were analyzed by read-based core-genome SNP comparison and within-colony variant allele-frequency screening. (B) Pairwise SNP distances among the nine colonies in the shared callable core. All displayed comparisons were 0 SNP. (C) Per-colony sequencing depth, the number of high-priority intermediate-frequency SNVs, and the *bla*NDM-1 state. Blue and red indicate *bla*NDM-1*-positive* and *bla*NDM-1*-negative* colonies, respectively. VAF, variant allele frequency; SNV, single-nucleotide variant.

### Repeat-associated *bla*NDM-1 module loss in reassembled plasmids

In the reassemble data assemblies, Pkp02_1 and Pkp04_1 were 253,885 bp and 243,402 bp, respectively. Both were IncHI1B/IncFIB(K) multireplicon plasmids; neither was typeable by the current IncHI1 pMLST scheme and no IncF RST profile was assigned. Pkp04_1 lacked a 10,483-bp interval present in Pkp02_1. The interval was delimited by two directly oriented, identical 3116-bp repeats containing *qacEΔ1* and *sul1*, and Pkp04_1 retained one corresponding repeat at the junction. Long-read alignments, flanking Illumina depth, an independently generated Flye assembly, and three independently sequenced KP04 colonies supported the deleted structure. These data support a repeat-associated plasmid deletion and reduce the likelihood of an assembly artifact; they do not identify the molecular pathway responsible for the rearrangement (Fig. 3; Supplementary Table S9).

**Fig. 3:**
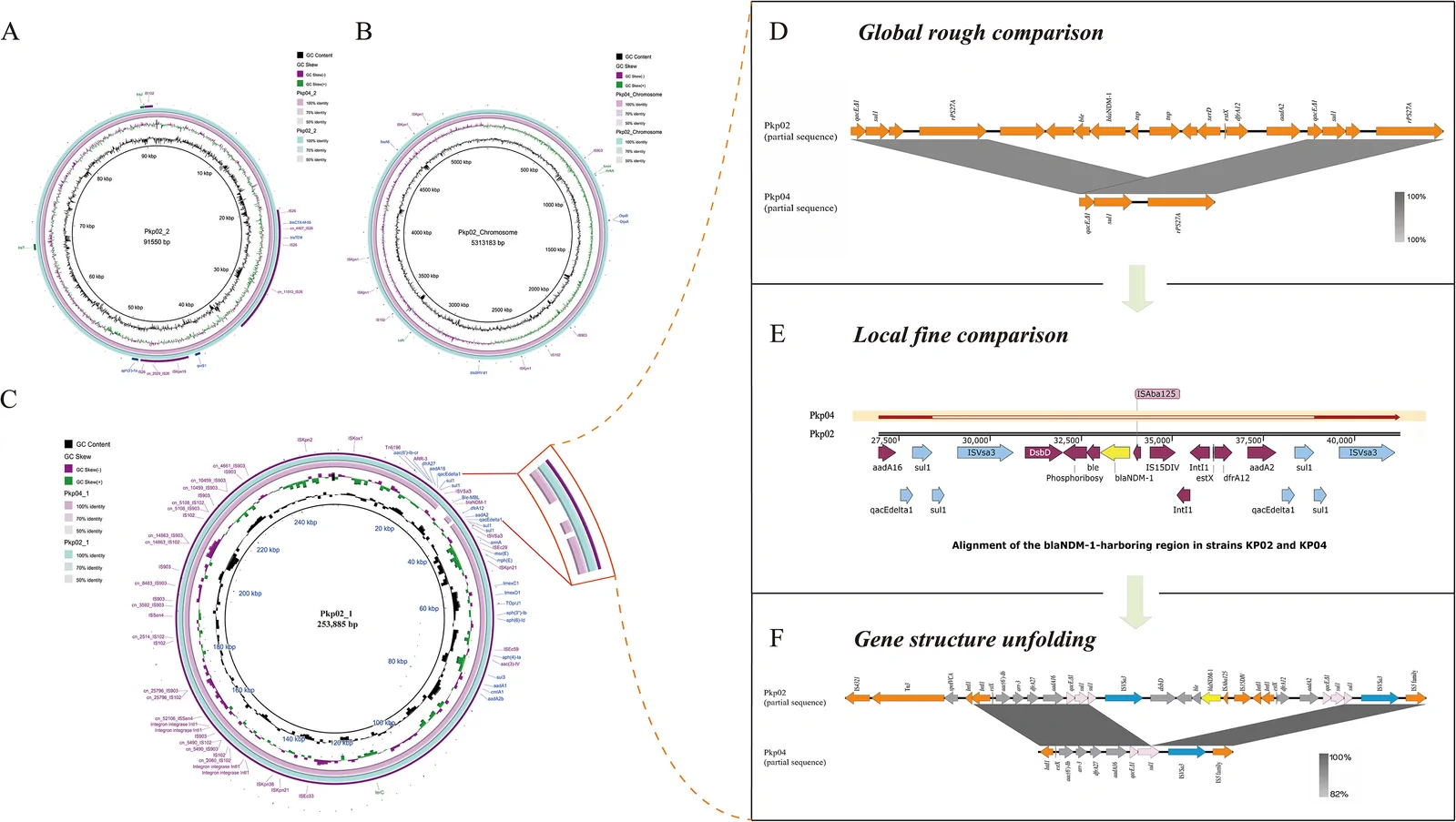
Plasmid, chromosome, and local structural comparisons of KP02 and KP04. (A) Circular comparison of the shorter plasmids Pkp02_2 and Pkp04_2. (B) Circular comparison of the KP02 and KP04 chromosomes. (C) Circular comparison of the reassembled plasmids Pkp02_1 and Pkp04_1. (D) Global alignment of the corresponding plasmid sequences. (E) Enlarged comparison of the *bla*NDM-1*-harboring* region. (F) Gene-level comparison showing the approximately 10.483-kb interval present in Pkp02_1 and absent from Pkp04_1, with a repeat-associated junction retained in Pkp04_1. Gray ribbons indicate homologous alignment blocks; the identity scale ranges from 82% to 100%.

### Phenotypic diversification during serial passage

Under antibiotic-free serial passage, two colony morphotypes became distinguishable at passage 10. KP02-G10-R formed larger colonies and retained carbapenem resistance, whereas KP02-G10-S formed smaller colonies and was carbapenem susceptible (Fig. 4A). Inhibition zones for carbapenems increased during serial passage, and KP02-G10-S showed restored carbapenem susceptibility. The representative MIC and disk-diffusion results and the complete susceptibility-panel profile are shown in Fig. 4.

**Fig. 4:**
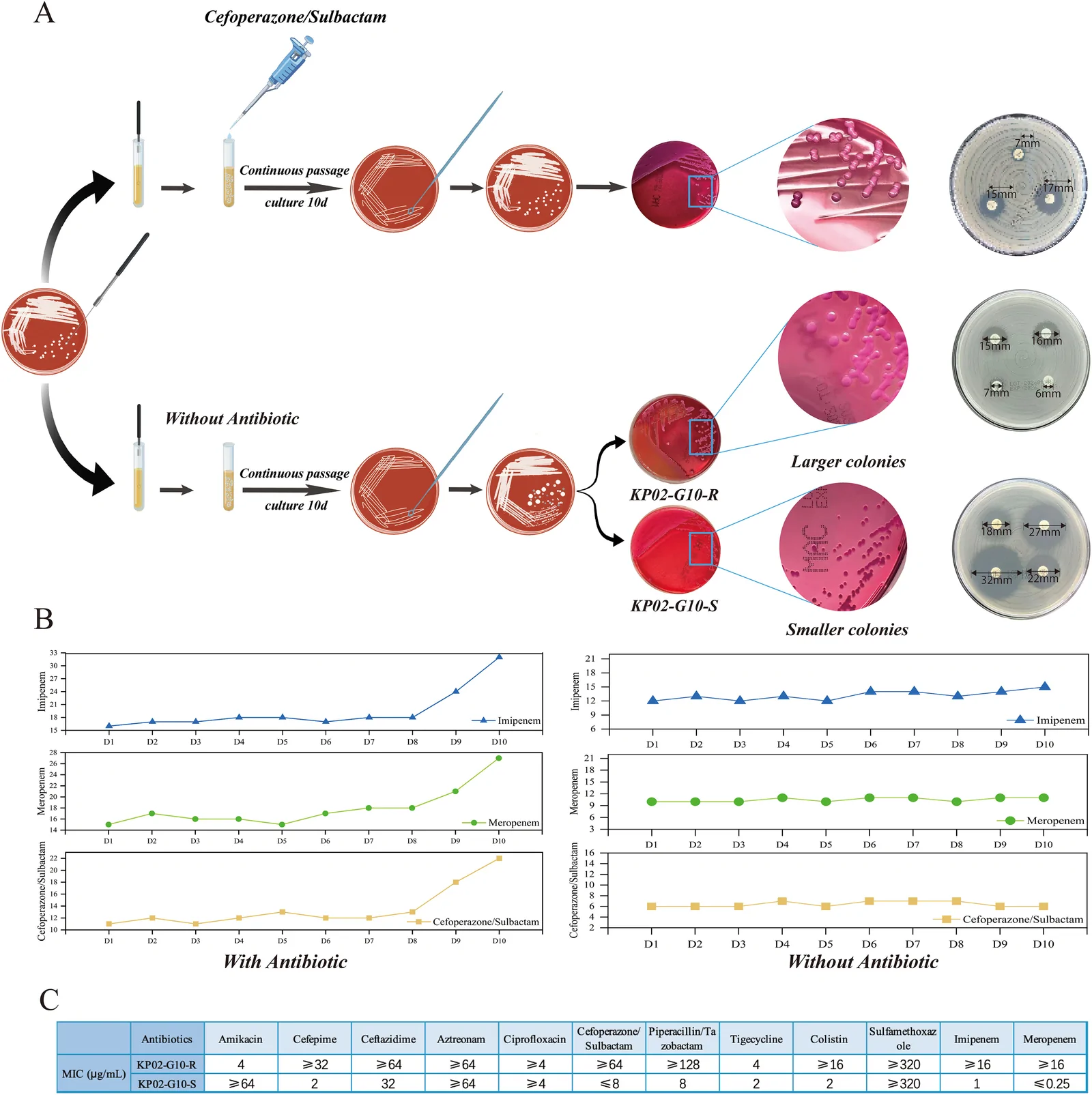
Phenotypic diversification during serial passage of KP02. (A) KP02 was passaged for 10 days in broth containing a subinhibitory concentration of cefoperazone–sulbactam or in antibiotic free broth. Two colony types appeared in the antibiotic free culture on day 10. KP02-G10-R formed larger colonies and remained carbapenem resistant. KP02-G10-S formed smaller colonies and became carbapenem susceptible. Colony morphology and disk diffusion results are shown. (B) Inhibition zone diameters for imipenem, meropenem, and cefoperazone–sulbactam were recorded from day 1 to day 10 under both conditions. (C) Minimum inhibitory concentrations of KP02-G10-R and KP02-G10-S. MIC indicates minimum inhibitory concentration.

### Molecular validation of *bla*NDM-1 module loss and candidate circular intermediate

Structural PCR and Sanger sequencing supported the presence of the *bla*NDM-1-containing region in KP02 and its absence in KP04. An outward-facing PCR product was detected in KP02 at the expected size for the 5804-bp amplicon predicted from a 10,483-bp candidate circular model; no corresponding product was detected in KP04. The purified product was assembled as a 5804-bp sequence matching the predicted circular path, and bidirectional Sanger reads crossed the modeled junction. These data support a sequence-verified candidate circular intermediate, while the abundance and complete 10,483-bp circular molecule remain unresolved. RT-qPCR shown in Fig. 5E detected target-specific *bla*NDM-1 amplification in KP02, KP03, KP02-G10, and KP02-G10-R. KP04 and KP02-G10-S were classified as target not detected because their signals were undetermined or late and nonspecific. For graphical display, target-not-detected wells were represented by a baseline 2^-ΔΔCt value of 1; the corresponding bars do not indicate detected *bla*NDM-1 transcripts. The raw Ct values and the final assay calls are reported in Supplementary Table S16. An additional five-isolate RT-qPCR run provided concordant supplementary confirmation (Supplementary Fig. S4).

**Fig. 5:**
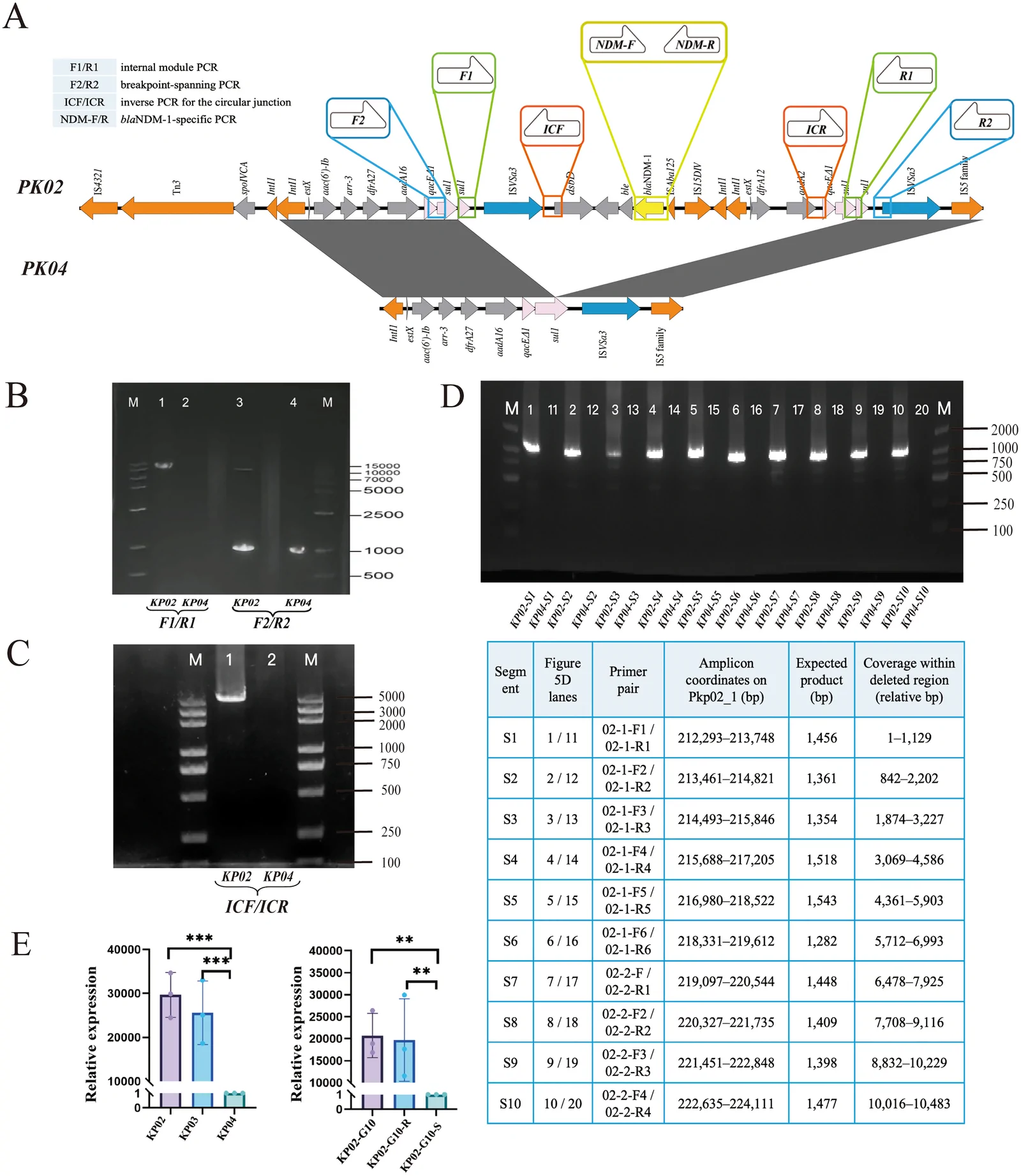
Molecular validation of *bla*NDM-1 module loss and a candidate circular intermediate. (A) Schematic of the targeted region and the locations of the F1/R1, F2/R2, ICF/ICR, and NDM-F/NDM-R primer pairs. Gray shading indicates homologous sequences. F1 and R1 amplify the internal region of the module. F2 and R2 amplify across the deletion junction. The outward facing primers ICF2 and ICR2 detect the candidate circular junction. NDM-F and NDM-R specifically detect *bla*NDM-1. (B) Structural PCR validation. Lanes 1 and 2 show F1/R1 products from KP02 and KP04, respectively, and lanes 3 and 4 show F2/R2 products from KP02 and KP04, respectively. F1/R1 interrogates the module-containing interval and yields the expected 10,375-bp product in KP02, with no corresponding complete product in KP04. F2/R2 interrogates the repeat-associated structure: KP02 has predicted 1549-bp and 12,032-bp product boundaries, whereas KP04 has the 1549-bp product from the retained repeat-associated region. (C) Outward-facing ICF2/ICR2 PCR. A product compatible with the predicted 5804-bp amplicon from the candidate circular model was detected in KP02, whereas no corresponding product was detected in KP04. (D) Segmented PCR of the deleted region using ten overlapping primer pairs. Lanes 1 to 10 contain the S1 to S10 products from KP02, and lanes 11 to 20 contain the corresponding reactions from KP04. The lanes are displayed as paired reactions from lanes 1 and 11 through lanes 10 and 20. All expected products were detected in KP02, whereas no visible products were detected in the corresponding KP04 lanes. The overlapping amplicons provide continuous coverage of the entire 10,483 bp region. S1 and S10 extend into the left and right flanking sequences, respectively, and the individual product lengths therefore should not be added. Primer pairs, amplicon coordinates, and expected product sizes are listed in the accompanying table. (E) Original RT-qPCR data. The left panel compares KP02, KP03, and KP04; the right panel compares KP02-G10, KP02-G10-R, and KP02-G10-S. Expression was normalized to *rpoB* and calculated with the 2^-ΔΔCt method. Each point represents one of three independent RNA extractions, and bars show mean ± SD. For graphical display, target-not-detected wells were represented by a baseline 2^-ΔΔCt value of 1; the corresponding bars do not indicate detected *bla*NDM-1 transcripts. The raw Ct values, final calls, and an additional five-isolate confirmatory run are provided in Supplementary Table S16 and Supplementary Fig. S4.

### Antibiotic-free fitness advantage associated with the module-lacking plasmid state

Under antibiotic-free conditions, KP04 showed higher growth and final biomass than KP02 (Fig. 6). During the 4-h competition assay, the meropenem-resistant fraction declined rapidly; because differential plating does not provide a lineage-invariant marker, this observation is supportive rather than definitive lineage-specific evidence. Conjugation yielded *bla*NDM-1-positive *E. coli* J53 transconjugants, supporting transferability of the KP02 plasmid. KP02 and KP04 produced comparable dose-dependent mortality profiles in *Galleria mellonella* across the tested inocula (Supplementary Figs. S1 and S3). Together, these findings show an association between absence of the resistance module and improved fitness under antibiotic-free conditions (Supplementary Table S11).

**Fig. 6:**
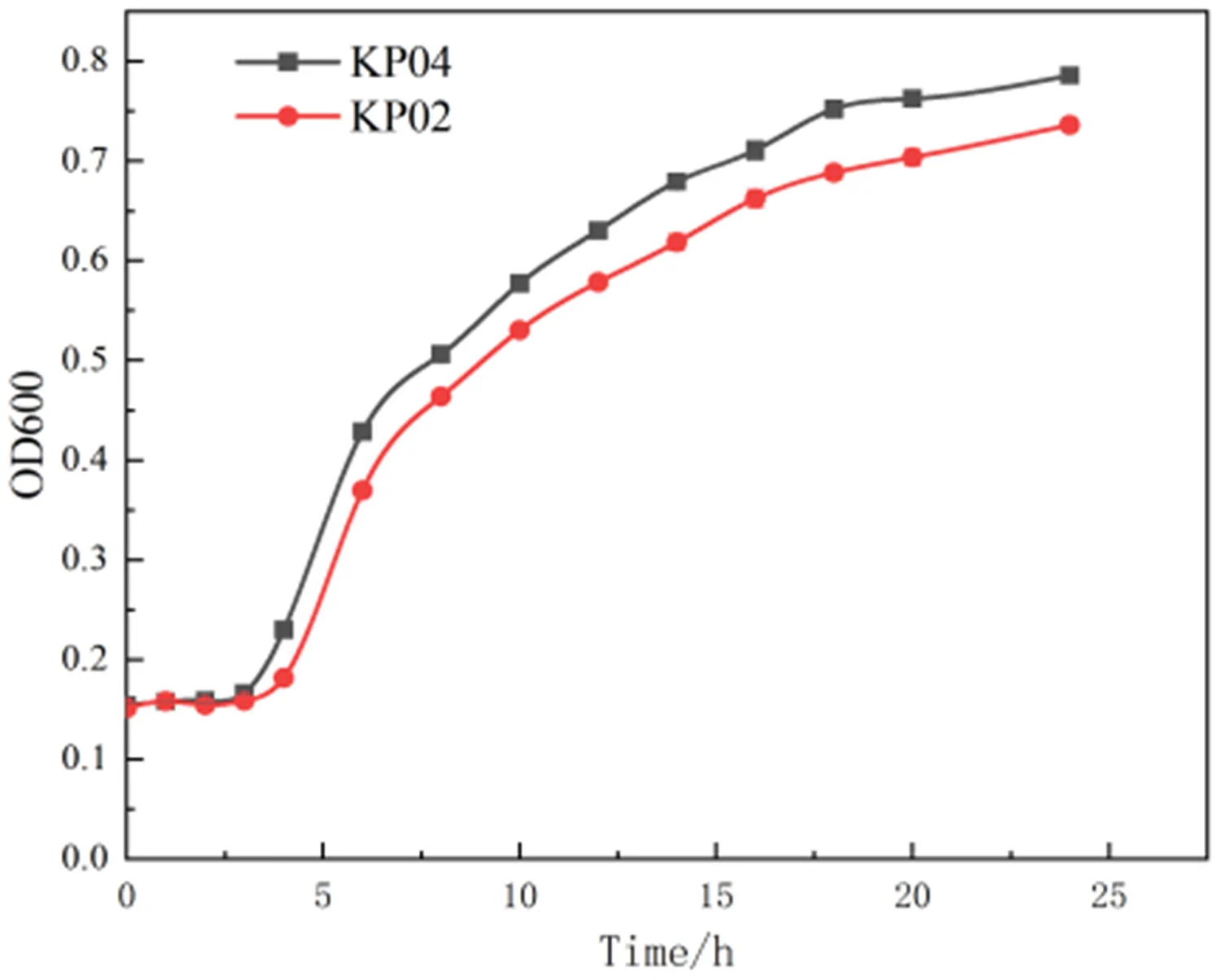
Growth comparison of KP02 and KP04 under antibiotic-free conditions.

Growth curves showing OD600 over time for KP02 and KP04. KP04 reached a higher final biomass under the tested antibiotic-free conditions; the comparison is presented as an association and does not assign the difference uniquely to the deleted interval.

### Distribution of direct-repeat architectures among complete *bla*NDM-1-positive plasmids

A contextual comparison of complete *bla*NDM-1-positive plasmid sequences showed related directly repeated *sul1*–*qacEΔ1*–IS*Vsa3*-associated regions in diverse plasmid backgrounds (Fig. 7). The comparison places the Pkp02_1 structure in a broader genomic context but does not estimate prevalence, deletion frequency, or circularization frequency.

**Fig. 7:**
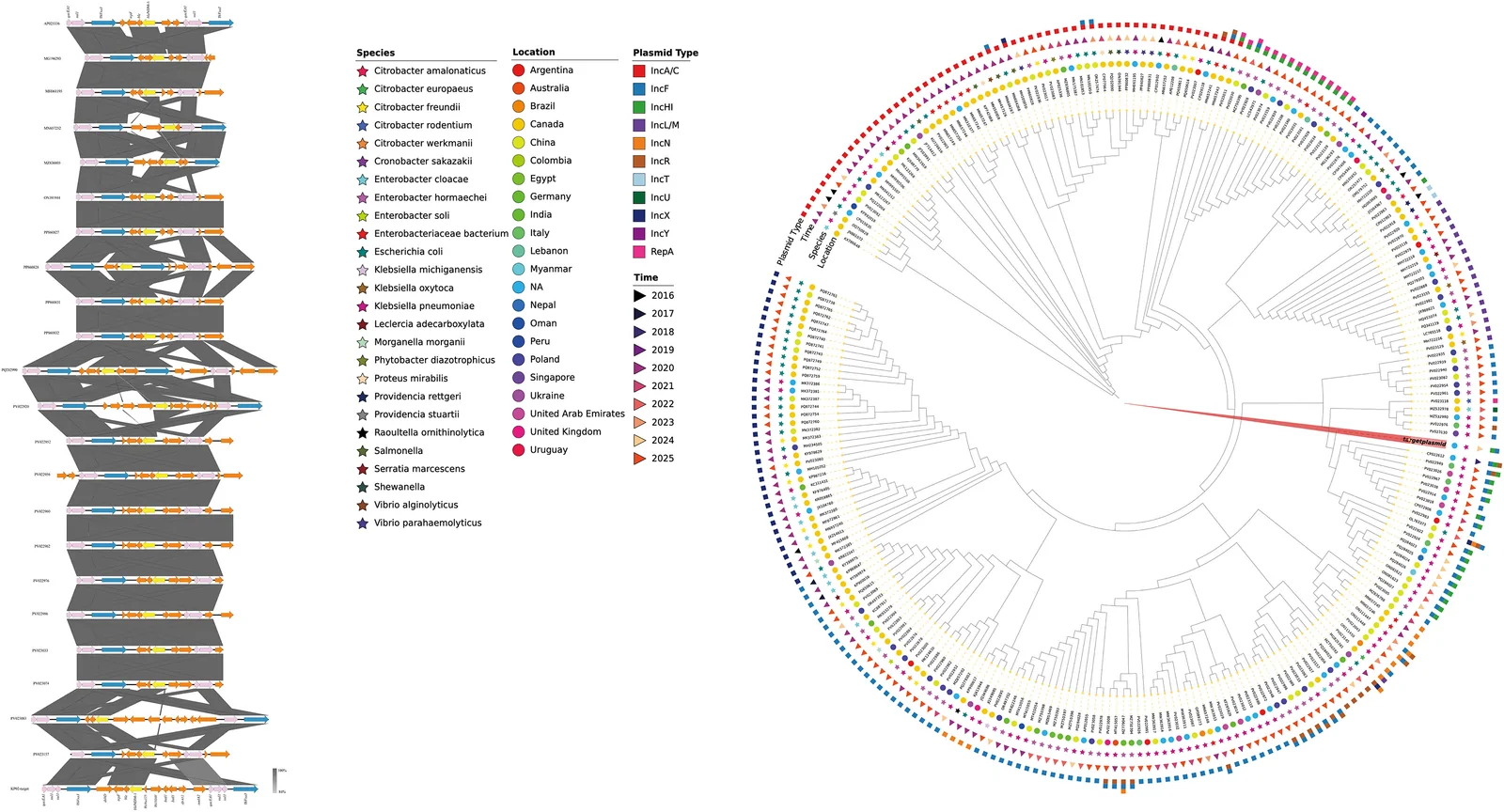
Distribution of directly repeated *sul1*–*qacEΔ1*–IS*Vsa3* architectures among complete *bla*NDM-1*-positive* plasmids. (A) Comparative synteny of representative complete *bla*NDM-1-positive plasmids carrying directly repeated *sul1–qacEΔ1*–IS*Vsa3* regions. Yellow arrows indicate *bla*NDM-1, and gray shading indicates homologous regions. (B) Mash-based relationship tree of the complete *bla*NDM-1-positive plasmids included in the contextual comparison. Branches corresponding to related repeat architectures are highlighted; annotations indicate plasmid type, species, geographic origin, and isolation year. This analysis describes structural context and does not estimate the prevalence or mechanism of deletion.

## Discussion

This case links carbapenem phenotypic heterogeneity to a defined plasmid structural difference within a closely related ST37/KL104 *Klebsiella pneumoniae* lineage. KP03 and KP04 were recovered from the same primary culture, and independent-colony sequencing showed that the resistant and susceptible phenotypes represented stable major genomic states. The loss of the *bla*NDM-1-containing region in Pkp04_1 therefore provides a genomic explanation for the susceptible phenotype, rather than a susceptibility result alone. The observation extends reports of within-patient plasmid dynamics and reversion to carbapenem susceptibility in *Klebsiella pneumoniae* (Villa et al., 2013; Stohr et al., 2020). It also highlights a practical diagnostic issue: selection of a single colony can under-sample phenotypic diversity, particularly when resistance is heterogeneous or transiently silent (Pereira et al., 2021; Lin et al., 2024; Göttig et al., 2016; Qin et al., 2024; Wagner et al., 2023).

The 10,483-bp difference in this study was bounded by directly repeated *sul1*-*qacEΔ1*-containing regions. *bla*NDM genes commonly occur in complex resistance regions that combine transposon-associated segments, class 1 integron remnants, and repeated sequences (Tavares et al., 2022; Liu et al., 2025; Pereira et al., 2021). Such architecture creates opportunities for local rearrangement and changes in resistance-gene content. Here, reassembled plasmids, independent assembly, long-read mapping, depth analysis, segmented PCR, and Sanger sequencing all supported two plasmid configurations. The 5804-bp outward-facing PCR product further supports a candidate circular configuration and provides a testable model for excision of the resistance module during plasmid remodeling.

The phenotypic experiments place the structural finding in a selection context. Under antibiotic-free serial passage, KP02 generated resistant and susceptible derivatives after ten passages, and KP04 reached a higher final biomass in antibiotic-free medium. Fitness trade-offs associated with plasmid-borne resistance and resistance-gene dosage have been described in *Klebsiella pneumoniae* and other pathogens (Pal and Andersson, 2024; Cho et al., 2025; Göttig et al., 2016). Our findings support the hypothesis that reduced antibiotic pressure can enrich module-lacking variants when their growth advantage outweighs the benefit of maintaining carbapenem resistance. This balance may help explain why closely related isolates with contrasting phenotypes can emerge during prolonged infection or laboratory passage.

The contextual plasmid comparison places this case within a broader structural setting and identifies related directly repeated *sul1*–*qacEΔ1*–IS*Vsa3*-associated configurations across diverse plasmid backgrounds. Together with the conjugative transfer of the KP02 plasmid, this observation suggests that repeat-associated resistance regions merit surveillance in diverse plasmid backgrounds (Yang et al., 2021; Wu et al., 2019). Long-read plasmid-resolved sequencing and multi-colony sampling are particularly valuable when discordant colony morphologies or antimicrobial susceptibility results are observed. These approaches can identify structural variants that are difficult to infer from a single consensus genome or a single colony.

Two limitations define the causal scope of this study. The unavailable original wound material precludes reconstruction of the initial subpopulation frequencies and temporal order of diversification. Genetic perturbation and isogenic reconstruction will be required to identify the molecular pathway responsible for the deletion and to quantify the module-specific component of the fitness difference. The documented coexistence of stable plasmid states, however, provides a clinically grounded model for investigating resistance-module instability in larger longitudinal collections.

## Conclusion

In this patient-derived ST37/KL104 lineage, a repeat-associated *bla*NDM-1 module deletion coincided with carbapenem phenotypic diversification. The data provide a clinically grounded model for testing in broader collections.

## Ethics approval

Ethics approval for this study was obtained from the Institutional Ethics Committee of the China–Japan 10.13039/501100008235Union Hospital of Jilin University (No. 2026,032,603).

## Funding

This work was supported by the Provincial Science and Technology Program (YDZJ202402064CXJD) and the Provincial Health Commission Key Research Project (2025SCZ21).

## CRediT authorship contribution statement

**Meiling Jiao:** Conceptualization, Methodology, Investigation, Data curation, Formal analysis, Visualization, Writing – original draft. **Sibo Wang:** Conceptualization, Methodology, Investigation, Data curation, Formal analysis, Visualization, Writing – original draft. **Qingyu Meng:** Investigation, Resources, Data curation. **Hao Liu:** Investigation, Data curation. **Juntian Jiang:** Investigation, Data curation. **Xuesong Xu:** Supervision, Project administration, Funding acquisition, Writing – review & editing. **Chao Yan:** Supervision, Project administration, Funding acquisition, Writing – review & editing.

## Declaration of competing interest

The authors declare that they have no known competing financial interests or personal relationships that could have appeared to influence the work reported in this paper.

## Data Availability

Raw sequencing data generated in this study have been deposited in the NCBI BioProject database under accession number PRJNA1463666, with associated datasets and metadata available through the Sequence Read Archive and BioSample databases. The annotated reassemble data Pkp02_1 and Pkp04_1 plasmid sequences have been submitted to GenBank under submission tracking identifiers SUB16355010, SUB16357637, SUB16355053, and SUB16357641; final accession numbers will be added when released. The initial and reassemble data sequence versions, lengths, and GenBank submission identifiers are documented in Supplementary Table S13.

## Appendix Supplementary materials

Image, application 1
