# Supplementary material for: Within-patient carbapenem heteroresistance in klebsiella pneumoniae associated with a repeat-associated blaNDM-1 resistance-module deletion
Source: Curr Res Microb Sci. 2026 Aug 29;11:100659. doi: 10.1016/j.crmicr.2026.100659 (PMC13632529; doi:10.1016/j.crmicr.2026.100659)

Revised Supplementary Appendix

Contents

Supplementary Methods....2
Supplementary Table S1. Clinical provenance and primary culture characteristics....3
Supplementary Table S2. Antimicrobial susceptibility results....3
Supplementary Table S3. Sequencing quality control....4
Supplementary Table S4. Cohort-wide shared-core result and secondary pairwise callable-site audit....5
Supplementary Table S5. Within-colony VAF summary....6
Supplementary Table S6. *bla*NDM-1 status in independent colonies....6
Supplementary Table S7. Institutional lineage context....7
Supplementary Table S8. ST37 genomic distance....7
Supplementary Table S9. Plasmid deletion architecture....8
Supplementary Table S10. Circular intermediate evidence....8
Supplementary Table S11. Fitness comparison evidence....9
Supplementary Table S12. Extraction, sequencing, and PCR reagents....9
Supplementary Table S13. Plasmid sequence availability....10
Supplementary Table S14. Plasmid replicon and pMLST typing....10
Supplementary Table S15. PCR assays, primer sequences, lanes and expected products....10
Supplementary Table S16. Figure 5E and confirmatory RT-qPCR raw Ct values and assay calls....14
Supplementary Figures S1-S4....22

Supplementary Methods

Independent-colony sequencing and variant screening

Three independently picked colonies from KP02, KP03 and KP04 were sequenced separately. Independent-colony libraries were prepared with the Rapid Plus DNA Lib Prep Kit for Illumina (RK20208) and sequenced in PE150 mode on a NovaSeq X Plus Series platform (Illumina, San Diego, CA, USA). Reads were trimmed with Trimmomatic 0.38, mapped to the fixed reassemble data KP02 chromosome, and analyzed with Snippy 4.6.0 and snp-dists 1.2.0. VAF candidates were recounted with quality, depth, allele-count and strand-support filters.

Plasmid structural validation

Corrected reassemble data assemblies were generated with Flye 2.9.6 and polished with Polypolish 0.6.1. Long-read CIGAR evidence, Illumina depth, an independent Flye assembly and independent KP04 colonies were used to audit the approximately 10.483-kb deletion.

PCR and Sanger validation

Structural PCR, segmented PCR, outward-facing PCR and Sanger sequencing were performed using the reagents and primer pairs listed in Tables S12 and S15. Expected sizes were calculated from the reassemble data sequence or, for the outward-facing assay, the candidate circular model. Each Figure 5 assay is linked to its available lane, primer, product, and sequencing record.

Candidate circular intermediate

The 10,483-bp candidate circular model predicts a 5,804-bp outward-facing product. The purified product was assembled from Sanger reads and bidirectional junction reads. Endpoint PCR was interpreted qualitatively and was not used to infer abundance.

RT-qPCR reporting

The RT-qPCR experiments displayed in Figure 5E used total RNA extracted with the Omega E.Z.N.A. Bacterial RNA Kit R6950-01, reverse transcription with FastKing RT Kit with gDNase KR116, and ChamQ Universal SYBR qPCR Master Mix Q711 with *rpoB* as the reference gene. Each isolate was analyzed using three independent RNA extractions, each measured in technical triplicate. For graphical display, target-not-detected wells were represented by a baseline $2^{-\Delta\Delta Ct}$ value of 1; these values are not interpreted as *bla*NDM-1 transcripts. A separate five-isolate RT-qPCR run performed in 2026 used three technical wells per target and is included only as confirmatory supplementary evidence in Supplementary Figure S4. In both datasets, late nonspecific or control-overlapping signals were recorded as target not detected.

Fitness, conjugation and Galleria assays

Growth and competition procedures followed the revised Methods. Conjugation and Galleria mellonella assays are summarized in the main text, with source images and detailed procedures retained in the supplementary evidence package.

Supplementary Table S1

Clinical provenance and primary culture characteristics of KP02, KP03 and KP04.

Item	KP02	KP03	KP04
Identification number	24112075	24112181-2	24112181-1
Sampling date and time	2024-11-01	2024-11-05	2024-11-05
Specimen type	Pus and wound secretion	Pus and wound secretion	Pus and wound secretion
Anatomical site of the wound	Right lower limb	Right lower limb	Right lower limb
Original culture request form and microbiology report	See figure	See figure	See figure
Whether the two colonies were recovered from the same primary culture plate	-	Yes	Yes
Culture medium	Columbia blood agar	Columbia blood agar	Columbia blood agar
Number of colonies selected from the primary culture plate	1	1	1
Rationale for selecting the two colonies, such as differences in colony morphology or antimicrobial susceptibility phenotype	-	Larger, whitish colony	Smaller, grayish colony
All strains were primary clinical isolates stored in 25% glycerol without serial passage	All strains were primary clinical isolates stored in 25% glycerol without serial passage	All strains were primary clinical isolates stored in 25% glycerol without serial passage	All strains were primary clinical isolates stored in 25% glycerol without serial passage

Supplementary Table S2

Antimicrobial susceptibility results for KP02, KP03 and KP04.

Antibiotics	KP02	KP03	KP04
Amikacin	>=64.0	>=64.0	>=64.0
Cefepime	>=32.0	>=32.0	2.0
Ceftazidime	>=64.0	>=64.0	16.0
Aztreonam	>=64.0	>=64.0	16.0
Ciprofloxacin	>=4.0	>=4.0	>=4.0
Cefoperazone/sulbactam	32.0	>=64.0	<=8.0
Piperacillin/tazobactam	>=128.0	>=128.0	<=4.0
Tigecycline	>=8.0	-	-

Antibiotics	KP02	KP03	KP04
Colistin	>=16.0	-	-
Imipenem	>=16.0	>=16.0	<=0.25
Meropenem	>=16.0	>=16.0	<=0.25
Levofloxacin (LVX)	>=8.0	>=8.0	>=8.0
Tobramycin (TOB)	>=16.0	>=16.0	>=16.0
Doxycycline (DOX)	8.0	8	4.0
Minocycline (MNO)	>=16.0	>=16.0	>=16.0
Trimethoprim/sulfamethoxazole (SXT)	>=320.0	>=320.0	>=320.0
Ticarcillin/clavulanate	>=128.0	>=128.0	<=8.0
Ceftriaxone	6	6	6
Cefuroxime	6	6	6
Cefazolin	6	6	6
Ampicillin/sulbactam	6	6	15

Supplementary Table S3. Sequencing QC

sample_id	parent_isolate	clean_read_pairs	clean_Q30_rate	chromosome_mean_depth	chromosome_coverage_percent	assembly_total_bp	assembly_contigs	assembly_N50	species	sequence_type
KP02-1	KP02	3666916	0.98654561	148.426	99.9998	5576563	84	348919	Klebsiella pneumoniae	37
KP02-2	KP02	3181345	0.98618009	132.102	99.9998	5575586	87	310490	Klebsiella pneumoniae	37
KP02-3	KP02	3266629	0.98621203	134.608	99.9999	5578267	81	431825	Klebsiella pneumoniae	37
KP03-1	KP03	3278699	0.98630362	134.416	99.9999	5576301	86	310490	Klebsiella pneumoniae	37
KP03-2	KP03	2812139	0.98625135	116.798	99.9992	5573758	90	249346	Klebsiella pneumoniae	37
KP03-3	KP03	2955437	0.98608599	119.118	99.9992	5576486	84	310491	Klebsiella pneumoniae	37
KP04-1	KP04	2959948	0.9863179	123.842	99.9989	5573253	76	348919	Klebsiella pneumoniae	37
KP04-2	KP04	2770905	0.98618155	114.966	99.9988	5572773	77	348751	Klebsiella pneumoniae	37
KP04-3	KP04	3132495	0.98644556	130.978	99.9988	5570613	81	310490	Klebsiella pneumoniae	37

Supplementary Table S4. Cohort-wide shared-core result and secondary pairwise callable-site audit

Interpretive note. Figure 2B reports the cohort-wide shared callable core, which retains only positions called unambiguously in all nine colonies. All pairwise distances in that analysis were 0 SNP. The table below reports a secondary pairwise callable-site audit, in which a site is retained when it is callable in the two samples being compared. The six 1-SNP entries involving KP03-1 or KP03-2 and KP04-1, KP04-2, or KP04-3 all arise from the same locally variable site. This site was excluded from the cohort-wide core because it was not callable across all nine colonies. These entries therefore represent one site observed across six pairwise comparisons rather than six independent SNPs.

sample_1	sample_2	pairwise_callable_sites	pairwise_SNP_distance
KP02-1	KP02-2	5253591	0
KP02-1	KP02-3	5253573	0
KP02-1	KP03-1	5253692	0
KP02-1	KP03-2	5253308	0
KP02-1	KP03-3	5253404	0
KP02-1	KP04-1	5253405	0
KP02-1	KP04-2	5253310	0
KP02-1	KP04-3	5253605	0
KP02-2	KP02-3	5253477	0
KP02-2	KP03-1	5253500	0
KP02-2	KP03-2	5253280	0
KP02-2	KP03-3	5253290	0
KP02-2	KP04-1	5253297	0
KP02-2	KP04-2	5253194	0
KP02-2	KP04-3	5253441	0
KP02-3	KP03-1	5253570	0
KP02-3	KP03-2	5253249	0
KP02-3	KP03-3	5253308	0
KP02-3	KP04-1	5253271	0
KP02-3	KP04-2	5253182	0
KP02-3	KP04-3	5253451	0
KP03-1	KP03-2	5253391	0

sample_1	sample_2	pairwise_callable_sites	pairwise_SNP_distance
KP03-1	KP03-3	5253488	0
KP03-1	KP04-1	5253485	1
KP03-1	KP04-2	5253337	1
KP03-1	KP04-3	5253607	1
KP03-2	KP03-3	5253237	0
KP03-2	KP04-1	5253267	1
KP03-2	KP04-2	5253078	1
KP03-2	KP04-3	5253351	1
KP03-3	KP04-1	5253281	0
KP03-3	KP04-2	5253120	0
KP03-3	KP04-3	5253367	0
KP04-1	KP04-2	5253445	0
KP04-1	KP04-3	5253656	0
KP04-2	KP04-3	5253530	0

Supplementary Table S5. Within-colony VAF summary

sample_id	evaluated_sites	sites_with_AF_0.05_to_0.80	excluded_sites	high_priority_SNV_candidates
KP02-1	11	4	4	0
KP02-2	11	4	4	0
KP02-3	11	5	5	0
KP03-1	11	5	5	0
KP03-2	11	5	5	0
KP03-3	11	5	5	0
KP04-1	11	2	2	0
KP04-2	11	3	3	0
KP04-3	11	4	4	0

Supplementary Table S6. *bla*NDM-1 status in independent colonies

sample_id	parent_isolate	*bla*NDM1_detected	module_to_flank_depth_ratio	*bla*NDM1_to_flank_depth_ratio	*bla*NDM1_assembly_coverage_percent	*bla*NDM1_identity_percent	structure_call

sample_id	parent_isolate	*bla*NDM1_detected	module_to_flank_depth_ratio	*bla*NDM1_to_flank_depth_ratio	*bla*NDM1_assembly_coverage_percent	*bla*NDM1_identity_percent	structure_call
KP02-1	KP02	yes	0.6234	1.06433	100	100	retained_module_supported
KP02-2	KP02	yes	0.68923	1.18476	100	100	retained_module_supported
KP02-3	KP02	yes	0.70369	1.19189	100	100	retained_module_supported
KP03-1	KP03	yes	0.7041	1.24771	100	100	retained_module_supported
KP03-2	KP03	yes	0.67705	1.18965	100	100	retained_module_supported
KP03-3	KP03	yes	1.46588	2.65709	100	100	retained_module_supported
KP04-1	KP04	no	0.01421	0	0	0	deleted_module_supported
KP04-2	KP04	no	0.01787	0	0	0	deleted_module_supported
KP04-3	KP04	no	0.01502	0	0	0	deleted_module_supported

Supplementary Table S7. Institutional lineage context

Isolate ID	Collection period	Relation to index department	Sequence type	Analysis role
K13	2024-10 to 2024-11	outside index department	ST11	contemporaneous CRKP context
K14	2024-10 to 2024-11	outside index department	ST11	contemporaneous CRKP context
K15	2024-10 to 2024-11	outside index department	ST11	contemporaneous CRKP context
K16	2024-10 to 2024-11	outside index department	ST11	contemporaneous CRKP context
K17	2024-10 to 2024-11	outside index department	ST15	contemporaneous CRKP context
K18	2024-10 to 2024-11	outside index department	ST11	contemporaneous CRKP context
K38	2024-10 to 2024-11	outside index department	ST11	contemporaneous CRKP context
K43	2024-10 to 2024-11	outside index department	ST11	contemporaneous CRKP context
KK167	2024	outside index department	ST37	broader 2024 assembly sensitivity comparator

Supplementary Table S8. ST37 genomic distance

Comparator	Index isolate	Sequence input	Pairwise callable positions	Pairwise differences
KK167	KP02	KK167 assembly and KP02 original reads	5053842	3018
KK167	KP03	KK167 assembly and KP03 original reads	5053856	3018
KK167	KP04	KK167 assembly and KP04 original reads	5053875	3018

Supplementary Table S9. Plasmid deletion architecture

Evidence	Sample or comparison	Reference coordinates or region	Quantitative result	Interpretation
Direct repeat A	Pkp02_1	212,852 to 215,967	3,116 bp	Contains transposase, sul1, and *qacEΔ1*
Direct repeat B	Pkp02_1	223,335 to 226,450	3,116 bp and 100% identical to repeat A	Directly oriented repeat flanking the resistance region
Repeat start spacing	Pkp02_1	212,852 to 223,335	10,483 bp	Equals the alignment defined deletion length
Alignment defined deletion	Pkp04 versus Pkp02_1	Pkp02_1 212,620 to 223,102	10,483 bp	*bla*NDM-1 resistance region absent from Pkp04_1
Retained junction repeat	Pkp04_1	57,961 to 61,076	3,116 bp and 100% identical to both Pkp02_1 repeats	One intact repeat remains in the deleted structure
ONT deletion support	KP04 mapped to Pkp02_1	Informative primary alignments with MAPQ at least 30	440 of 499 support a 10.4 to 10.6 kb deletion	Read level support for the deletion structure
Strict ONT endpoint support	KP04 mapped to Pkp02_1	Both endpoints within 250 bp of the alignment defined boundaries	16 of 499 informative alignments	Endpoint counts are lower because identical repeats permit equivalent placements
Illumina depth	KP04 mapped to Pkp02_1	Before, deleted interval, and after	349.00 fold, 6.88 fold, and 298.28 fold	Marked depletion within the deleted interval
Independent Flye assembly	KP04 versus KP02 Plasmid1	Independent ONT only closed assemblies	10,475 bp difference	Independent assembly supports the same structural change
Independent colony WGS	Three KP04 colonies	*bla*NDM-1 and module read depth plus assembly coverage	All three support the deleted module state	The major deleted structure was stable across independent colonies

Supplementary Table S10. Circular intermediate evidence

Evidence	Method	Result	Independent check	Interpretation
Source module sequence	Segment PCR and Sanger	11,744 bp nonredundant sequence	Identical to fixed reassemble data Pkp02_1 source region	Supports the complete source module sequence
Circle specific amplification	Outward primers on the linear plasmid	Predicted 5,804 bp product on the 10,483 bp circular model	KP02 band above 5,000 bp and no KP04 product	Supports a circle specific product
Complete inverse PCR product	Assembled Sanger contig	5,804 bp	Exact match to the predicted circular path	Argues against an unrelated smaller amplicon
Raw Sanger coverage	Ten AB1 chromatograms	5,682 of 5,804 bp or 97.90 percent	Only primer adjacent terminal bases were outside Q20 trimmed coverage	Supports the assembled product
Forward junction read	Independent Sanger read	951 bp at 100 percent identity	420 and 531 high quality bases on the two sides	Direct circular junction support
Reverse junction read	Independent Sanger read	966 bp at 100 percent identity	698 and 268 high quality bases on the two sides	Independent opposite direction junction support
KP04 deletion reads	ONT remapping	440 of 499 informative reads	16 reads met strict endpoint criteria	Read level deletion support

Evidence	Method	Result	Independent check	Interpretation
KP04 deletion depth	Illumina remapping	Mean depth 6.88 within the interval	Mean depth 349.00 and 298.28 in the flanks	Supports loss of the interval
Independent assembly	Flye assembly	10,475 bp concordant difference	Within 8 bp of the 10,483 bp reference interval	Reduces assembler dependence
Circular abundance	Validated junction quantitative assay	Not measured	No copy number or frequency inferred from endpoint PCR	Conclusion restricted to qualitative detection

Supplementary Table S11. Fitness comparison evidence

Evidence	Design	Result	Supported interpretation
Independent colony core SNP analysis	Three colonies each from KP02, KP03, and KP04	No mutually differing high confidence SNPs in the shared callable core	The clinical isolates are extremely closely related
Resistance module state	Three independent KP02 and three independent KP04 colonies	All KP02 colonies retained *bla*NDM-1 and all KP04 colonies supported module deletion	The contrasting module states were stable across independently selected colonies
Serial passage time scale	Daily passage of KP02 without antibiotics	Resistant and susceptible derivatives were recovered after ten passages	The 4 h competition interval was substantially shorter than the observed passage period
Independent growth phenotype	Separate antibiotic free monoculture growth curves	KP04 showed higher OD600 values and final biomass than KP02	This observation does not depend on differential plating during coculture
Competition phenotype	Phenotype based differential plating during 4 h coculture	The carbapenem resistant fraction declined rapidly	Supportive evidence with unresolved lineage assignment for any newly susceptible KP02 cells

Supplementary Table S12. Extraction, sequencing, and PCR reagents

Workflow	Use	Product and catalog number	Manufacturer
Original whole genome sequencing	Genomic DNA extraction	Omega Bacterial DNA Kit (D3350)	OMEGA
Independent-colony whole genome sequencing	Illumina library preparation	Rapid Plus DNA Lib Prep Kit for Illumina (RK20208)	Manufacturer not specified in available record
Independent-colony whole genome sequencing	Paired-end sequencing	NovaSeq X Plus Series PE150	Illumina, San Diego, CA, USA
Structural PCR and Sanger validation	Genomic DNA extraction	Omega Bacterial DNA Kit (D3350)	OMEGA
Plasmid electroporation	Plasmid DNA purification	QIAGEN Plasmid Midi Kit	QIAGEN
RT qPCR	Total RNA extraction	E.Z.N.A. Bacterial RNA Kit (R6950-01)	Omega Bio-tek
Reverse transcription	cDNA synthesis with genomic DNA removal	FastKing RT Kit with gDNase (KR116)	Tiangen Biotech
Quantitative PCR	SYBR quantitative PCR	ChamQ Universal SYBR qPCR Master Mix (Q711)	Vazyme
Segment PCR	Segment and nested PCR amplification	2× EasyTaq PCR SuperMix with dye	TransGen Biotech

Workflow	Use	Product and catalog number	Manufacturer
Inverse PCR	Long range inverse PCR amplification	2× MegaPfu Premix with dye (AS231)	TOLOBIO
PCR product purification	Agarose gel extraction	Omega Gel Extraction Kit (D2500)	OMEGA
Gel electrophoresis	Agarose gel preparation	AgaroseLE (AgaroseLE)	Kumei Biotechnology

Supplementary Table S13. Plasmid sequence availability

Plasmid	Strain	Sequence version	Length bp	Submission ID	GenBank accession	Use in revision
Pkp02_1	KP02	Initial assembly	253843	SUB16355010	Pending assignment	Version tracking
Pkp02_1	KP02	Corrected reassemble data	253885	SUB16357637	Pending assignment	Used in revised analyses
Pkp04_1	KP04	Initial assembly	243363	SUB16355053	Pending assignment	Version tracking
Pkp04_1	KP04	Corrected reassemble data	243402	SUB16357641	Pending assignment	Used in revised analyses

Supplementary Table S14. Plasmid replicon and pMLST typing

Plasmid	IncHI1B marker	Coverage percent	Identity percent	IncFIB(K) marker	Coverage percent	Identity percent	Replicon classification	IncHI1 pMLST	IncF RST
Pkp02_1	IncHI1B_1_pNDM-MAR	100.00	100.00	IncFIB(K)_1_Kpn3	100.00	91.07	IncHI1B/IncFIB(K)	Not typeable	No RST assigned
Pkp04_1	IncHI1B_1_pNDM-MAR	100.00	100.00	IncFIB(K)_1_Kpn3	100.00	91.07	IncHI1B/IncFIB(K)	Not typeable	No RST assigned

Supplementary Table S15. PCR assays, primer sequences, lanes and expected products

Figure or assay	Lane	Sample	Primer pair	Expected product	Observed result	Interpretation or confirmation
Figure 5B	1	KP02	F1 and R1	10,375 bp	Band at approximately 10 kb	Intact resistance-module long-range product
Figure 5B	2	KP04	F1 and R1	No corresponding complete product	No band	Full module target is absent after deletion
Figure 5B	3	KP02	F2 and R2	1,549 bp and 12,032 bp	Strong approximately 1.5 kb band and faint long band	Repeat-associated products from related primer sites
Figure 5B	4	KP04	F2 and R2	1,549 bp	Band at approximately 1.5 kb	Retained sul1-related structure after deletion
Figure 5C	KP02	KP02	ICF2 and ICR2	5,804 bp	Band above the 5,000 bp marker	Complete 5,804-bp Sanger product and bidirectional junction reads
Figure 5C	KP04	KP04	ICF2 and ICR2	No circle-specific product	No corresponding band	Negative structural comparison

Figure or assay	Lane	Sample	Primer pair	Expected product	Observed result	Interpretation or confirmation
Supplementary Figure S2 panel A	NTC	No template control	KP04 breakpoint out F and R	No product	No band	Negative control
Supplementary Figure S2 panel A	KP04	KP04	KP04 breakpoint out F and R	1,549 bp	Band at approximately 1.5 kb	Bidirectional Sanger assembly yielded a 1,437 bp junction contig
Supplementary Figure S2 panel B	KP02	KP02	ICF2 and ICR2	5,804 bp	Band above the 5,000 bp marker	Complete 5,804 bp Sanger product and bidirectional junction reads
Supplementary Figure S2 panel B	KP04	KP04	ICF2 and ICR2	No circle specific product	No corresponding band	Negative structural comparison
Source region validation, evidence archive	Segment 1	KP02	02-1-F and 02-1-R	7,417 bp shortest intended product	Lane identity not recoverable from the supplied unlabelled gel	Used as a Sanger sequencing template and excluded as a stand alone gel claim
Source region validation, evidence archive	Segment 2	KP02	02-2-F and 02-2-R	7,768 bp shortest intended product	Lane identity not recoverable from the supplied unlabelled gel	Used as a Sanger sequencing template and excluded as a stand alone gel claim

Figure 5D lane map. The physical order is M, 1, 11, 2, 12, 3, 13, through 10, 20, M. Lanes 1–10 contain KP02 DNA and lanes 11–20 contain KP04 DNA. The paired lanes 1/11 through 10/20 correspond in order to entries 3 through 12 of the PCR/Sanger service record. Thus, pair 1/11 corresponds to entry 3 and pair 10/20 corresponds to entry 12. The displayed positive bands are in the KP02 lanes; no visible product was observed in the paired KP04 lanes. The complete primer sequences are listed in the molecular-validation primer table below.

The service-record entry numbers are retained for traceability. The primer names and sequences in Table S15 are the primary identifiers for the current Figure 5D paired-lane map.

Supplementary Table S15 continued. Segmented PCR paired-lane map

Service record entry	Figure 5D lanes	Primer pair	KP02 expected (bp)	KP02 observed	KP04 expected (bp)	KP04 observed	Sanger record
3	1 / 11	02-1-F1 / 02-1-R1	1,456	Lane 1, approximately 1.5 kb	1,456	Lane 11, no visible product	Overlapping KP02 source-module reads
4	2 / 12	02-1-F2 / 02-1-R2	1,361	Lane 2, approximately 1.4 kb	1,361	Lane 12, no visible product	Overlapping KP02 source-module reads
5	3 / 13	02-1-F3 / 02-1-R3	1,354	Lane 3, faint approximately 1.4 kb	1,354	Lane 13, no visible product	Overlapping KP02 source-module reads
6	4 / 14	02-1-F4 / 02-1-R4	1,518	Lane 4, approximately 1.5 kb	No correctly oriented product	Lane 14, no visible product	Overlapping KP02 source-module reads
7	5 / 15	02-1-F5 / 02-1-R5	1,543	Lane 5, approximately 1.5 kb	No correctly oriented product	Lane 15, no visible product	Overlapping KP02 source-module reads
8	6 / 16	02-1-F6 / 02-1-R6	1,282	Lane 6, approximately 1.3 kb	No correctly oriented product	Lane 16, no visible product	Overlapping KP02 source-module reads
9	7 / 17	02-2-F / 02-2-R1	1,448	Lane 7, approximately 1.4 kb	No correctly oriented product	Lane 17, no visible product	Overlapping KP02 source-module reads
10	8 / 18	02-2-F2 / 02-2-R2	1,409	Lane 8, approximately 1.4 kb	No correctly oriented product	Lane 18, no visible product	Overlapping KP02 source-module reads
11	9 / 19	02-2-F3 / 02-2-R3	1,398	Lane 9, approximately 1.4 kb	No correctly oriented product	Lane 19, no visible product	Overlapping KP02 source-module reads
12	10 / 20	02-2-F4 / 02-2-R4	1,477	Lane 10, approximately 1.5 kb	No correctly oriented product	Lane 20, no visible product	Overlapping KP02 source-module reads

Supplementary Table S15 continued. Molecular-validation primer sequences

Assay	Purpose	Primer name	Sequence 5 prime to 3 prime	Record status
Figure 5B structural PCR	Module-internal assay	F1	TCTTACCTGAAATTCCCTCACTCG	Confirmed and used
Figure 5B structural PCR	Module-internal assay	R1	ACCGGTAAACTGAGCCAATGAC	Confirmed and used
Figure 5B structural PCR	Repeat-associated structural assay	F2	AACAACGAGTTAATGAAGCCTTCTA	Confirmed and used
Figure 5B structural PCR	Repeat-associated structural assay	R2	CCAGGTCTTGAGTATCGTCTATCGT	Confirmed and used
Figure 5C inverse PCR	Candidate circular-intermediate amplification	ICF2	TTCCGTACACCCACTCCGTTTA	Confirmed and used
Figure 5C inverse PCR	Candidate circular-intermediate amplification	ICR2	AGTCGGGAAAATCTAACGTGCG	Confirmed and used
Figure 5D segmented PCR	Service record entry 3, lanes 1/11	02-1-F1	ATCAATGACCAAGTGAGCCA	Confirmed and used
Figure 5D segmented PCR	Service record entry 3, lanes 1/11	02-1-R1	ACTTACGATGAGCTGAATACGC	Confirmed and used

Assay	Purpose	Primer name	Sequence 5 prime to 3 prime	Record status
Figure 5D segmented PCR	Service record entry 4, lanes 2/12	02-1-F2	GGCGTGTAGCGAGAAACCAG	Confirmed and used
Figure 5D segmented PCR	Service record entry 4, lanes 2/12	02-1-R2	GACCGAGGGTTAGATCATGCC	Confirmed and used
Figure 5D segmented PCR	Service record entry 5, lanes 3/13	02-1-F3	AGCCCTCACGAGTTGTTATCA	Confirmed and used
Figure 5D segmented PCR	Service record entry 5, lanes 3/13	02-1-R3	GCATCGCATTTTATTTTCTTT	Confirmed and used
Figure 5D segmented PCR	Service record entry 6, lanes 4/14	02-1-F4	TGAGCCCCATACCTACAAAGC	Confirmed and used
Figure 5D segmented PCR	Service record entry 6, lanes 4/14	02-1-R4	CGGCATTTAGCTTTTCAGGC	Confirmed and used
Figure 5D segmented PCR	Service record entry 7, lanes 5/15	02-1-F5	TTAGAACCAGAAGCCAAAACG	Confirmed and used
Figure 5D segmented PCR	Service record entry 7, lanes 5/15	02-1-R5	AAGCTCTCGGGTAACATCAAG	Confirmed and used
Figure 5D segmented PCR	Service record entry 8, lanes 6/16	02-1-F6	TGAAGTGGTTCGCATCCTC	Confirmed and used
Figure 5D segmented PCR	Service record entry 8, lanes 6/16	02-1-R6	CTGATGGAGCTGCACATGAAC	Confirmed and used
Figure 5D segmented PCR	Service record entry 9, lanes 7/17	02-2-F	GTACTTAATCTGTCGGTGTTCAACG	Confirmed and used
Figure 5D segmented PCR	Service record entry 9, lanes 7/17	02-2-R1	TCCATACCGCCCATCTTGTC	Confirmed and used
Figure 5D segmented PCR	Service record entry 10, lanes 8/18	02-2-F2	GTCTGGCAGCACACTTCCTATC	Confirmed and used
Figure 5D segmented PCR	Service record entry 10, lanes 8/18	02-2-R2	CGTCGAACAAGATCAAGTCCG	Confirmed and used
Figure 5D segmented PCR	Service record entry 11, lanes 9/19	02-2-F3	GTGCGGTTACGTCGAATGTC	Confirmed and used
Figure 5D segmented PCR	Service record entry 11, lanes 9/19	02-2-R3	GCCGACGGCTATTACCTGTA	Confirmed and used
Figure 5D segmented PCR	Service record entry 12, lanes 10/20	02-2-F4	CGCAGCCCTGGTACTTCAC	Confirmed and used
Figure 5D segmented PCR	Service record entry 12, lanes 10/20	02-2-R4	GAAGACGATACGCTGACTCAATTAC	Confirmed and used
Source-module long-range PCR	First source region	02-1-F	CCTTGCCGTGTTTGCGAATTTT	Confirmed and used
Source-module long-range PCR	First source region	02-1-R	GCAAATAGTCGGTGGTGATAAACTT	Confirmed and used
Source-module long-range PCR	Second source region	02-2-F	GTACTTAATCTGTCGGTGTTCAACG	Confirmed and used
Source-module long-range PCR	Second source region	02-2-R	GCGGAATGTAGTGCTTACTTTGTC	Confirmed and used
Candidate-circle Sanger	Internal sequencing	WSB-ICF-F6	CGTGAGCTGCCACCAGAACG	Confirmed and used
Candidate-circle Sanger	Internal sequencing	WSB-ICF-R1	CTGGATAGAACACCAACCCG	Confirmed and used
Candidate-circle Sanger	Internal sequencing	WSB-PKP02-R0807	TTCGACGTCGCCGAAGAATT	Confirmed and used
Candidate-circle Sanger	Additional sequencing	WSB-1-R6-REV	TACGACGGGAGGAGAGATA	Confirmed and used
Candidate-circle Sanger	Additional sequencing	WSB-2-F4-F0728	GATCGAACAGGCTGGGAGTC	Confirmed and used
KP04 breakpoint PCR	Deletion-junction product	KP04 breakpoint out F	AACAACGAGTTAATGAAGCCTTCTA	Confirmed and used
KP04 breakpoint PCR	Deletion-junction product	KP04 breakpoint out R	CCAGGTCTTGAGTATCGTCTATCGT	Confirmed and used
RT-qPCR	*bla*NDM-1 expression	NDM-qF2	CCTATCTCGACATGCCGGGTT	Confirmed and used
RT-qPCR	*bla*NDM-1 expression	NDM-qR2	TCTCCTGCTTGATCCAGTTGAGG	Confirmed and used
RT-qPCR	rpoB reference	rpoB-qF2	TTCAGTACGATATCTACCGGCGTA	Confirmed and used
RT-qPCR	rpoB reference	rpoB-qR2	GGTGATAAGATGGCAGGTCGTC	Confirmed and used

Supplementary Table S16. Figure 5E and confirmatory RT-qPCR raw Ct values and assay calls

This table reports raw Ct values for the *blaNDM-1* target and the *rpoB* reference assay used for Figure 5E interpretation. Historical 16S measurements were not used for Figure 5E normalization and remain available in the submitted raw instrument records.

Dataset	Sample	Target	Independent RNA extraction	Technical repeat	Raw Ct or Cp	Tm 1 C	Tm 2 C	Genomic blaNDM-1	Final call
Original Figure 5E isolate assay	KP02	*rpoB*	1	1	24.4126224517822			Present	Detected reference
Original Figure 5E isolate assay	KP02	*rpoB*	1	2	23.4664039611816			Present	Detected reference
Original Figure 5E isolate assay	KP02	*rpoB*	1	3	23.098072052002			Present	Detected reference
Original Figure 5E isolate assay	KP02	*bla*NDM-1	1	1	20.3386516571045			Present	Detected
Original Figure 5E isolate assay	KP02	*bla*NDM-1	1	2	21.530143737793			Present	Detected
Original Figure 5E isolate assay	KP02	*bla*NDM-1	1	3	21.7540435791016			Present	Detected
Original Figure 5E isolate assay	KP04	*rpoB*	1	1	23.017915725708			Absent	Detected reference
Original Figure 5E isolate assay	KP04	*rpoB*	1	2	23.2726230621338			Absent	Detected reference
Original Figure 5E isolate assay	KP04	*rpoB*	1	3	23.5725841522217			Absent	Detected reference
Original Figure 5E isolate assay	KP04	*bla*NDM-1	1	1	Undetermined			Absent	Undetermined
Original Figure 5E isolate assay	KP04	*bla*NDM-1	1	2	37.030647277832			Absent	Late signal not quantified
Original Figure 5E isolate assay	KP04	*bla*NDM-1	1	3	36.2517967224121			Absent	Late signal not quantified
Original Figure 5E isolate assay	KP03	*rpoB*	1	1	20.652437210083			Present	Detected reference
Original Figure 5E isolate assay	KP03	*rpoB*	1	2	21.2292251586914			Present	Detected reference
Original Figure 5E isolate assay	KP03	*rpoB*	1	3	20.9265975952148			Present	Detected reference
Original Figure 5E isolate assay	KP03	*bla*NDM-1	1	1	18.240421295166			Present	Detected

Dataset	Sample	Target	Independent RNA extraction	Technical repeat	Raw Ct or Cp	Tm 1 C	Tm 2 C	Genomic blaNDM-1	Final call
Original Figure 5E isolate assay	KP03	*bla*NDM-1	1	2	19.6353912353516			Present	Detected
Original Figure 5E isolate assay	KP03	*bla*NDM-1	1	3	19.3798923492432			Present	Detected
Original Figure 5E isolate assay	KP02	*rpoB*	2	1	22.2569961547852			Present	Detected reference
Original Figure 5E isolate assay	KP02	*rpoB*	2	2	22.9298877716064			Present	Detected reference
Original Figure 5E isolate assay	KP02	*rpoB*	2	3	22.7484378814697			Present	Detected reference
Original Figure 5E isolate assay	KP02	*bla*NDM-1	2	1	19.7376327514648			Present	Detected
Original Figure 5E isolate assay	KP02	*bla*NDM-1	2	2	19.2899723052979			Present	Detected
Original Figure 5E isolate assay	KP02	*bla*NDM-1	2	3	19.4900188446045			Present	Detected
Original Figure 5E isolate assay	KP04	*rpoB*	2	1	21.3497200012207			Absent	Detected reference
Original Figure 5E isolate assay	KP04	*rpoB*	2	2	22.4253444671631			Absent	Detected reference
Original Figure 5E isolate assay	KP04	*rpoB*	2	3	22.3890762329102			Absent	Detected reference
Original Figure 5E isolate assay	KP04	*bla*NDM-1	2	1	32.8062858581543			Absent	Late signal not quantified
Original Figure 5E isolate assay	KP04	*bla*NDM-1	2	2	33.7146072387695			Absent	Late signal not quantified
Original Figure 5E isolate assay	KP04	*bla*NDM-1	2	3	37.0728874206543			Absent	Late signal not quantified
Original Figure 5E isolate assay	KP03	*rpoB*	2	1	19.691370010376			Present	Detected reference
Original Figure 5E isolate assay	KP03	*rpoB*	2	2	21.0970058441162			Present	Detected reference
Original Figure 5E isolate assay	KP03	*rpoB*	2	3	19.9271030426025			Present	Detected reference
Original Figure 5E isolate assay	KP03	*bla*NDM-1	2	1	17.9715347290039			Present	Detected

Dataset	Sample	Target	Independent RNA extraction	Technical repeat	Raw Ct or Cp	Tm 1 C	Tm 2 C	Genomic blaNDM-1	Final call
Original Figure 5E isolate assay	KP03	*bla*NDM-1	2	2	18.5653381347656			Present	Detected
Original Figure 5E isolate assay	KP03	*bla*NDM-1	2	3	18.6019191741943			Present	Detected
Original Figure 5E isolate assay	KP02	*rpoB*	3	1	22.8959636688232			Present	Detected reference
Original Figure 5E isolate assay	KP02	*rpoB*	3	2	22.7818298339844			Present	Detected reference
Original Figure 5E isolate assay	KP02	*rpoB*	3	3	22.5680313110352			Present	Detected reference
Original Figure 5E isolate assay	KP02	*bla*NDM-1	3	1	19.8009281158447			Present	Detected
Original Figure 5E isolate assay	KP02	*bla*NDM-1	3	2	19.6100788116455			Present	Detected
Original Figure 5E isolate assay	KP02	*bla*NDM-1	3	3	19.4271945953369			Present	Detected
Original Figure 5E isolate assay	KP04	*rpoB*	3	1	21.6866970062256			Absent	Detected reference
Original Figure 5E isolate assay	KP04	*rpoB*	3	2	21.7601451873779			Absent	Detected reference
Original Figure 5E isolate assay	KP04	*rpoB*	3	3	21.8576717376709			Absent	Detected reference
Original Figure 5E isolate assay	KP04	*bla*NDM-1	3	1	35.1085090637207			Absent	Late signal not quantified
Original Figure 5E isolate assay	KP04	*bla*NDM-1	3	2	34.091178894043			Absent	Late signal not quantified
Original Figure 5E isolate assay	KP04	*bla*NDM-1	3	3	31.9748783111572			Absent	Late signal not quantified
Original Figure 5E isolate assay	KP03	*rpoB*	3	1	21.1197948455811			Present	Detected reference
Original Figure 5E isolate assay	KP03	*rpoB*	3	2	21.2120304107666			Present	Detected reference
Original Figure 5E isolate assay	KP03	*rpoB*	3	3	21.2585792541504			Present	Detected reference
Original Figure 5E isolate assay	KP03	*bla*NDM-1	3	1	18.9189453125			Present	Detected

Dataset	Sample	Target	Independent RNA extraction	Technical repeat	Raw Ct or Cp	Tm 1 C	Tm 2 C	Genomic blaNDM-1	Final call
Original Figure 5E isolate assay	KP03	*bla*NDM-1	3	2	19.1944065093994			Present	Detected
Original Figure 5E isolate assay	KP03	*bla*NDM-1	3	3	18.7005958557129			Present	Detected
Historical passage assay	KP02-G10-R	*rpoB*	1	1	22.2917423248291			Present	Detected reference
Historical passage assay	KP02-G10-R	*rpoB*	1	2	22.2452526092529			Present	Detected reference
Historical passage assay	KP02-G10-R	*rpoB*	1	3	22.2231197357178			Present	Detected reference
Historical passage assay	KP02-G10-R	*bla*NDM-1	1	1	22.1004505157471			Present	Detected
Historical passage assay	KP02-G10-R	*bla*NDM-1	1	2	22.0126762390137			Present	Detected
Historical passage assay	KP02-G10-R	*bla*NDM-1	1	3	22.0568428039551			Present	Detected
Historical passage assay	KP02-G10-S	*rpoB*	1	1	20.683422088623			Absent	Detected reference
Historical passage assay	KP02-G10-S	*rpoB*	1	2	20.6495685577393			Absent	Detected reference
Historical passage assay	KP02-G10-S	*rpoB*	1	3	20.652250289917			Absent	Detected reference
Historical passage assay	KP02-G10-S	*bla*NDM-1	1	1	35.2671928405762			Absent	Late signal not quantified
Historical passage assay	KP02-G10-S	*bla*NDM-1	1	2	34.4457092285156			Absent	Late signal not quantified
Historical passage assay	KP02-G10-S	*bla*NDM-1	1	3	34.606761932373			Absent	Late signal not quantified
Historical passage assay	KP02-G10-R	*rpoB*	2	1	22.4490871429443			Present	Detected reference
Historical passage assay	KP02-G10-R	*rpoB*	2	2	22.4385070800781			Present	Detected reference
Historical passage assay	KP02-G10-R	*rpoB*	2	3	22.9262065887451			Present	Detected reference
Historical passage assay	KP02-G10-R	*bla*NDM-1	2	1	22.281717300415			Present	Detected
Historical passage assay	KP02-G10-R	*bla*NDM-1	2	2	22.2963638305664			Present	Detected
Historical passage assay	KP02-G10-	*bla*NDM-1	2	3	22.281661987			Present	Detected

Dataset	Sample	Target	Independent RNA extraction	Technical repeat	Raw Ct or Cp	Tm 1 C	Tm 2 C	Genomic blaNDM-1	Final call
	R				3047				
Historical passage assay	KP02-G10-S	*rpoB*	2	1	20.9295749664307			Absent	Detected reference
Historical passage assay	KP02-G10-S	*rpoB*	2	2	20.9587173461914			Absent	Detected reference
Historical passage assay	KP02-G10-S	*rpoB*	2	3	20.9128761291504			Absent	Detected reference
Historical passage assay	KP02-G10-S	*bla*NDM-1	2	1	35.953296661377			Absent	Late signal not quantified
Historical passage assay	KP02-G10-S	*bla*NDM-1	2	2	35.8946647644043			Absent	Late signal not quantified
Historical passage assay	KP02-G10-S	*bla*NDM-1	2	3	33.9303092956543			Absent	Late signal not quantified
Historical passage assay	KP02-G10-R	*rpoB*	3	1	23.2079753875732			Present	Detected reference
Historical passage assay	KP02-G10-R	*rpoB*	3	2	23.3038349151611			Present	Detected reference
Historical passage assay	KP02-G10-R	*rpoB*	3	3	23.4074382781982			Present	Detected reference
Historical passage assay	KP02-G10-R	*bla*NDM-1	3	1	19.9162273406982			Present	Detected
Historical passage assay	KP02-G10-R	*bla*NDM-1	3	2	19.9536552429199			Present	Detected
Historical passage assay	KP02-G10-R	*bla*NDM-1	3	3	19.8636493682861			Present	Detected
Historical passage assay	KP02-G10-S	*rpoB*	3	1	21.7414016723633			Absent	Detected reference
Historical passage assay	KP02-G10-S	*rpoB*	3	2	21.6991729736328			Absent	Detected reference
Historical passage assay	KP02-G10-S	*rpoB*	3	3	21.7983589172363			Absent	Detected reference
Historical passage assay	KP02-G10-S	*bla*NDM-1	3	1	32.2694129943848			Absent	Late signal not quantified
Historical passage assay	KP02-G10-S	*bla*NDM-1	3	2	33.7738418579102			Absent	Late signal not quantified
Historical passage assay	KP02-G10-S	*bla*NDM-1	3	3	33.727108001709			Absent	Late signal not quantified
Historical passage assay	KP02-G10	*rpoB*	1	1	21.966129303			Present	Detected reference

Dataset	Sample	Target	Independent RNA extraction	Technical repeat	Raw Ct or Cp	Tm 1 C	Tm 2 C	Genomic blaNDM-1	Final call
Historical passage assay	KP02-G10	*rpoB*	1	2	21.9667949677			Present	Detected reference
Historical passage assay	KP02-G10	*rpoB*	1	3	21.9078121185			Present	Detected reference
Historical passage assay	KP02-G10	*bla*NDM-1	1	1	22.5205478668			Present	Detected
Historical passage assay	KP02-G10	*bla*NDM-1	1	2	21.0155029297			Present	Detected
Historical passage assay	KP02-G10	*bla*NDM-1	1	3	22.0108127594			Present	Detected
Historical passage assay	KP02-G10	*rpoB*	2	1	20.9962005615			Present	Detected reference
Historical passage assay	KP02-G10	*rpoB*	2	2	21.980670929			Present	Detected reference
Historical passage assay	KP02-G10	*rpoB*	2	3	21.9962997437			Present	Detected reference
Historical passage assay	KP02-G10	*bla*NDM-1	2	1	22.3395023346			Present	Detected
Historical passage assay	KP02-G10	*bla*NDM-1	2	2	22.2170734406			Present	Detected
Historical passage assay	KP02-G10	*bla*NDM-1	2	3	22.1771507263			Present	Detected
Historical passage assay	KP02-G10	*rpoB*	3	1	22.3302364349			Present	Detected reference
Historical passage assay	KP02-G10	*rpoB*	3	2	22.4878196716			Present	Detected reference
Historical passage assay	KP02-G10	*rpoB*	3	3	22.9347820282			Present	Detected reference
Historical passage assay	KP02-G10	*bla*NDM-1	3	1	19.7435417175			Present	Detected
Historical passage assay	KP02-G10	*bla*NDM-1	3	2	19.9644851685			Present	Detected
Historical passage assay	KP02-G10	*bla*NDM-1	3	3	19.8956127167			Present	Detected
Additional 2026 confirmatory assay	KP02	*rpoB*	1	1	14	82.21		Present	Detected
Additional 2026 confirmatory assay	KP03	*rpoB*	1	1	14.25	82.26		Present	Detected
Additional 2026 confirmatory assay	KP04	*rpoB*	1	1	13.77	82.24		Absent	Detected
Additional 2026 confirmatory assay	KP02-G10-R	*rpoB*	1	1	13.28	82.23		Present	Detected
Additional 2026 confirmatory assay	KP02-G10-S	*rpoB*	1	1	12.71	82.21		Absent	Detected
Additional 2026 confirmatory assay	NTC	*rpoB*		1	30.7	80.43		NA	Control amplification
Additional 2026	KP02	*rpoB*	1	2	13.94	82.21		Present	Detected

Dataset	Sample	Target	Independent RNA extraction	Technical repeat	Raw Ct or Cp	Tm 1 C	Tm 2 C	Genomic blaNDM-1	Final call
confirmatory assay									
Additional 2026 confirmatory assay	KP03	*rpoB*	1	2	14.17	82.21		Present	Detected
Additional 2026 confirmatory assay	KP04	*rpoB*	1	2	13.75	82.21		Absent	Detected
Additional 2026 confirmatory assay	KP02-G10-R	*rpoB*	1	2	13.3	82.26		Present	Detected
Additional 2026 confirmatory assay	KP02-G10-S	*rpoB*	1	2	12.75	82.23		Absent	Detected
Additional 2026 confirmatory assay	NTC	*rpoB*		2	30.7	80.6		NA	Control amplification
Additional 2026 confirmatory assay	KP02	*rpoB*	1	3	14.07	82.21		Present	Detected
Additional 2026 confirmatory assay	KP03	*rpoB*	1	3	14.16	82.21		Present	Detected
Additional 2026 confirmatory assay	KP04	*rpoB*	1	3	13.66	82.21		Absent	Detected
Additional 2026 confirmatory assay	KP02-G10-R	*rpoB*	1	3	13.32	82.26		Present	Detected
Additional 2026 confirmatory assay	KP02-G10-S	*rpoB*	1	3	12.74	82.21		Absent	Detected
Additional 2026 confirmatory assay	NTC	*rpoB*		3	30.5	80.52		NA	Control amplification
Additional 2026 confirmatory assay	KP02	*bla*NDM-1	1	1	16.06	87.72		Present	Detected
Additional 2026 confirmatory assay	KP03	*bla*NDM-1	1	1	15.6	87.73		Present	Detected
Additional 2026 confirmatory assay	KP04	*bla*NDM-1	1	1	28.42	89.19		Absent	ND after specificity filtering
Additional 2026 confirmatory assay	KP02-G10-R	*bla*NDM-1	1	1	13.81	87.85		Present	Detected
Additional 2026 confirmatory assay	KP02-G10-S	*bla*NDM-1	1	1	26.06	88.98		Absent	ND after specificity filtering
Additional 2026 confirmatory assay	NTC	*bla*NDM-1		1	28.31	87.67		NA	Control amplification
Additional 2026 confirmatory assay	KP02	*bla*NDM-1	1	2	16.06	87.72		Present	Detected

Dataset	Sample	Target	Independent RNA extraction	Technical repeat	Raw Ct or Cp	Tm 1 C	Tm 2 C	Genomic blaNDM-1	Final call
Additional 2026 confirmatory assay	KP03	*bla*NDM-1	1	2	15.5	87.75		Present	Detected
Additional 2026 confirmatory assay	KP04	*bla*NDM-1	1	2	28.01	89.22		Absent	ND after specificity filtering
Additional 2026 confirmatory assay	KP02-G10-R	*bla*NDM-1	1	2	13.82	87.86		Present	Detected
Additional 2026 confirmatory assay	KP02-G10-S	*bla*NDM-1	1	2	26.25	88.97		Absent	ND after specificity filtering
Additional 2026 confirmatory assay	NTC	*bla*NDM-1		2	32.01	76.75	87.53	NA	Control amplification
Additional 2026 confirmatory assay	KP02	*bla*NDM-1	1	3	16.21	87.76		Present	Detected
Additional 2026 confirmatory assay	KP03	*bla*NDM-1	1	3	15.58	87.74		Present	Detected
Additional 2026 confirmatory assay	KP04	*bla*NDM-1	1	3	28.12	89.21		Absent	ND after specificity filtering
Additional 2026 confirmatory assay	KP02-G10-R	*bla*NDM-1	1	3	13.84	87.81		Present	Detected
Additional 2026 confirmatory assay	KP02-G10-S	*bla*NDM-1	1	3	28.51	88.27		Absent	ND after specificity filtering
Additional 2026 confirmatory assay	NTC	*bla*NDM-1		3	32.75	87.44		NA	Control amplification

Supplementary Figures

Supplementary Figure S1. Conjugation assay source image.

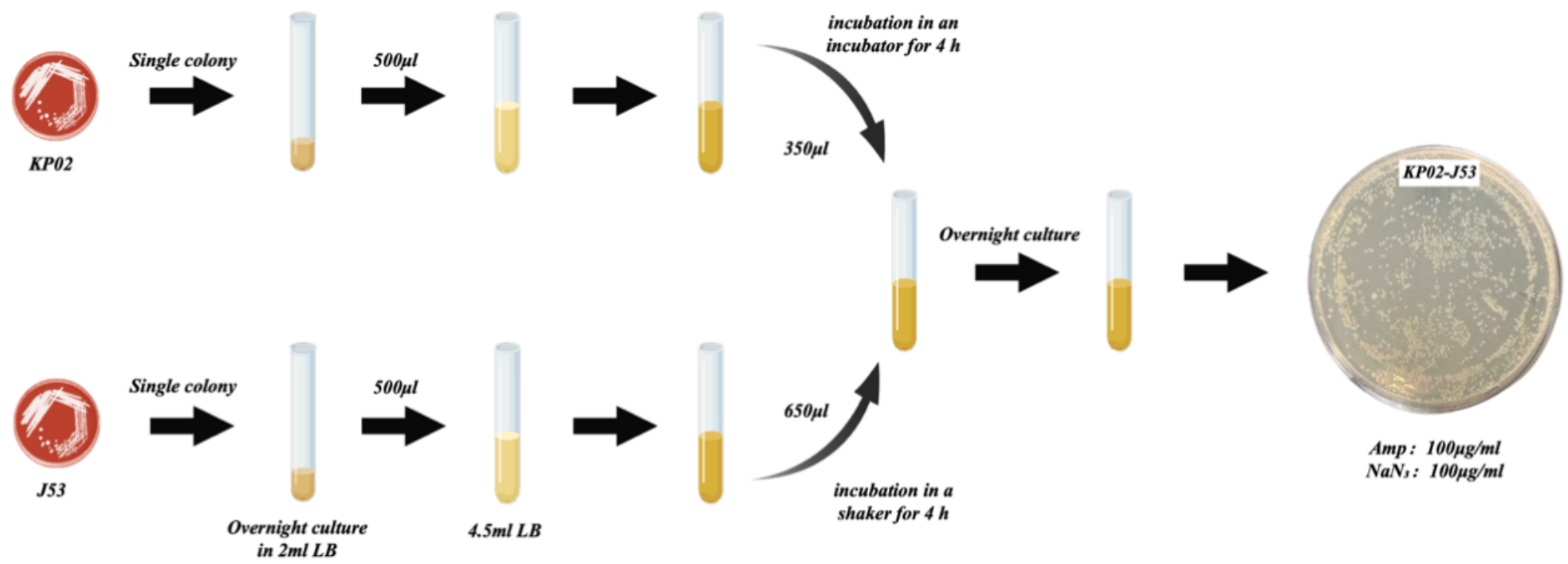

Supplementary Figure S2. KP04 breakpoint and candidate circular-intermediate PCR source images.

A KP04 breakpoint PCR

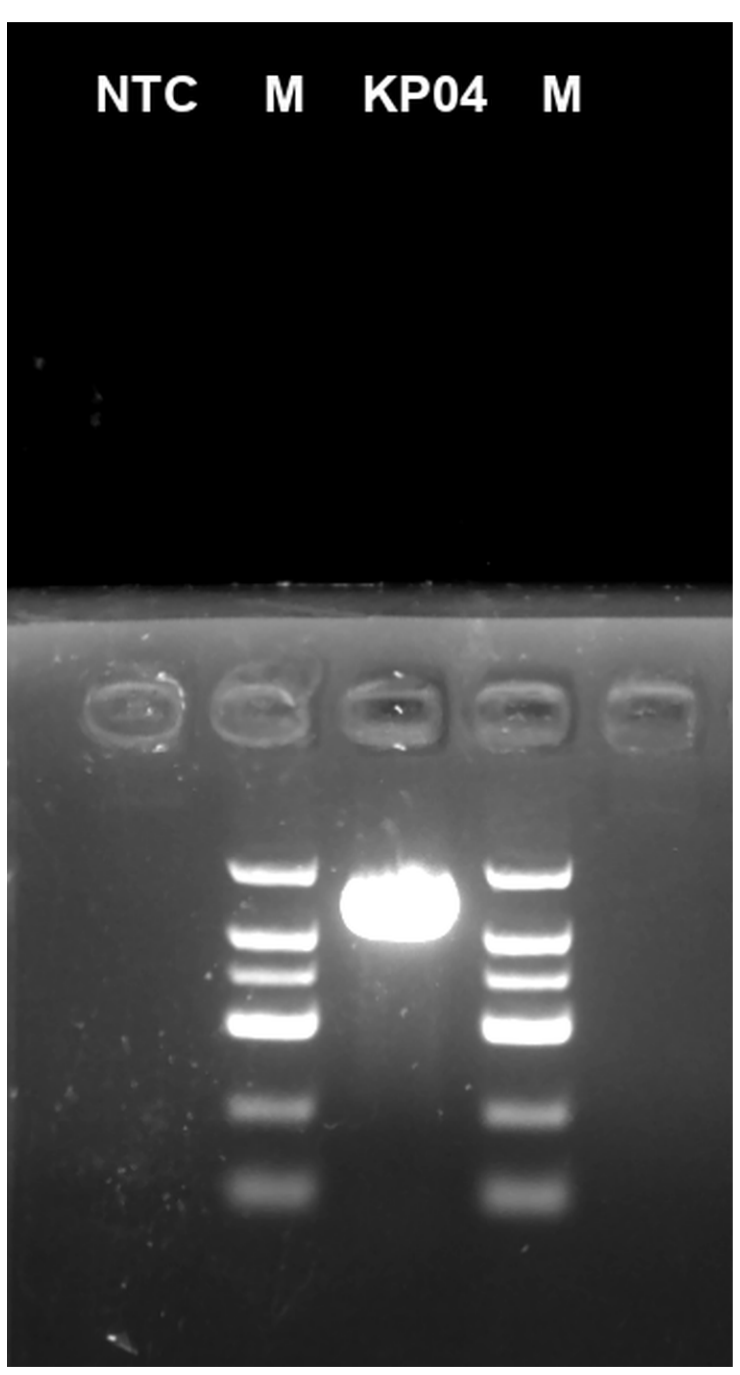

Supplementary Figure S3. Galleria mellonella infection assay source image.

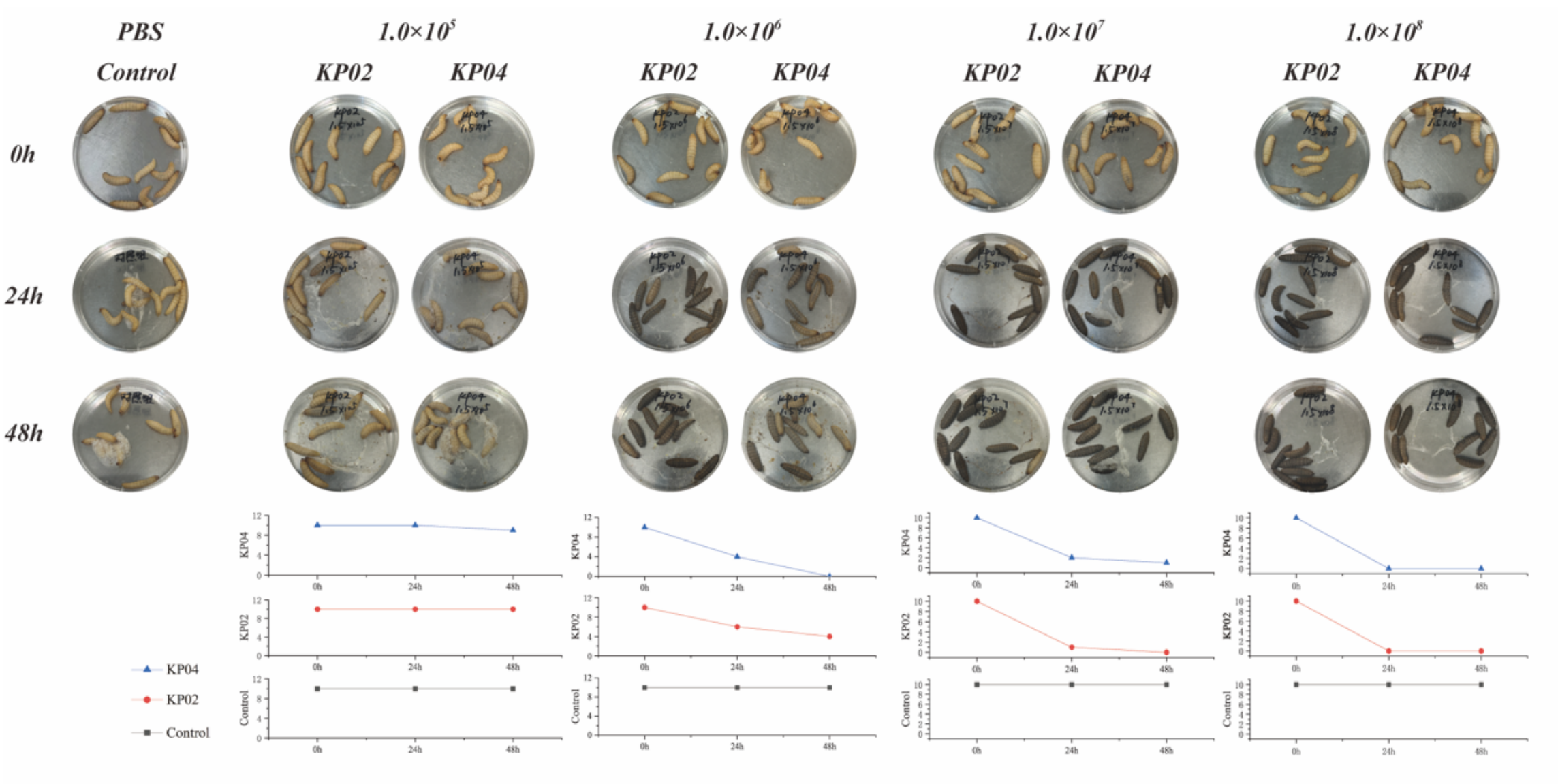

Supplementary Figure S4. Additional five-isolate RT-qPCR confirmation. The 2026 run was analyzed separately from the historical Figure 5E experiments. Relative *bla*NDM-1 expression was normalized to *rpoB* and calculated using the 2^-ΔΔCt method. The run used three technical wells per target. KP04 and KP02-G10-S were classified as target not detected after review of Ct values, melt curves, and no-template controls, and no relative-expression value was assigned to these samples. Bars show mean ± SD of technical wells. This figure is supplementary confirmatory evidence and does not replace the three-independent-RNA-extraction experiments in Figure 5E.

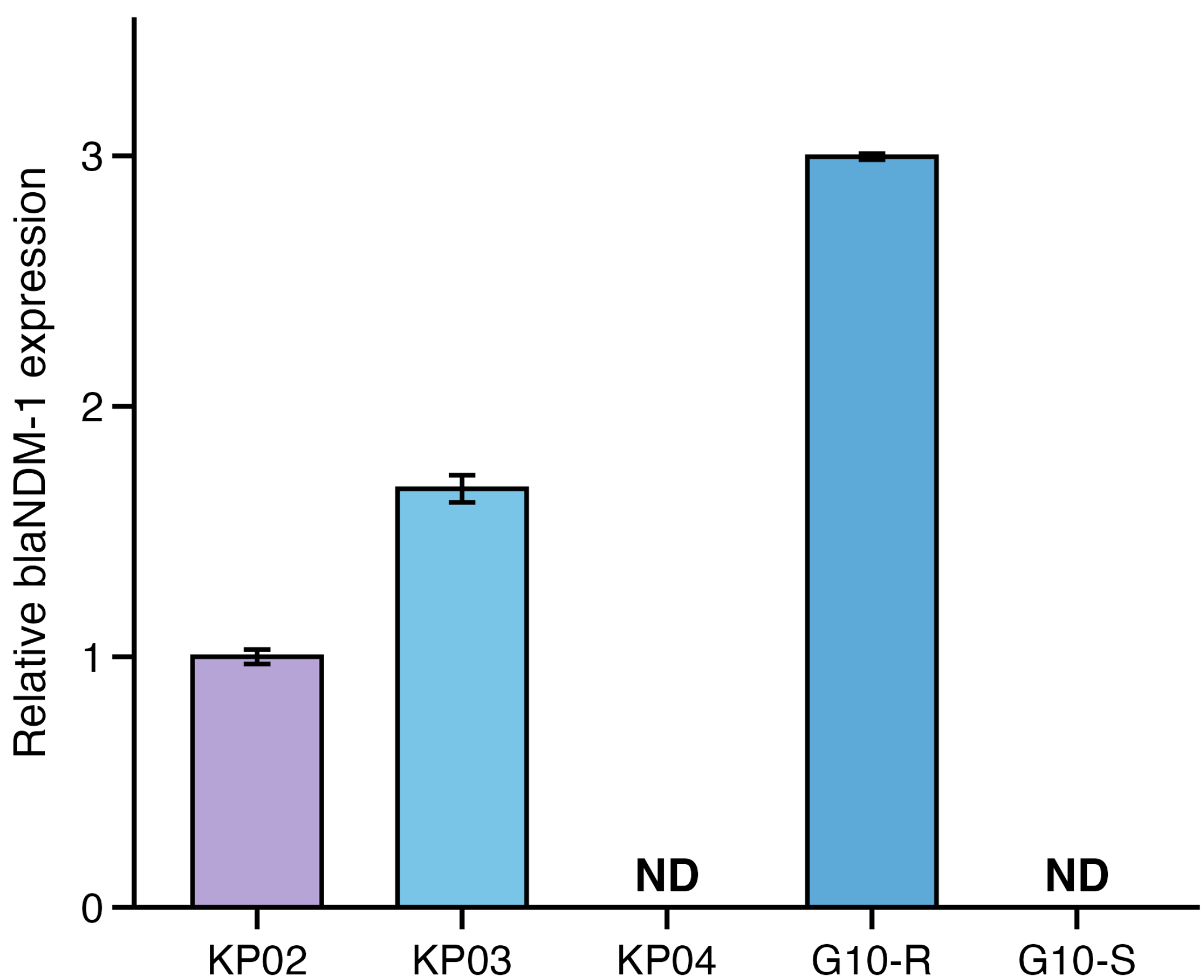

Supplement: Supplementary file 1 [file mmc1.pdf]
